# Exploration-Exploitation and Suicidal Behavior in Borderline Personality Disorder and Depression

**DOI:** 10.1001/jamapsychiatry.2024.1796

**Published:** 2024-07-10

**Authors:** Aliona Tsypes, Michael N. Hallquist, Angela Ianni, Aleksandra Kaurin, Aidan G. C. Wright, Alexandre Y. Dombrovski

**Affiliations:** 1 Department of Psychiatry, University of Pittsburgh School of Medicine, Pittsburgh, Pennsylvania; 2 Department of Psychology and Neuroscience, University of North Carolina, Chapel Hill; 3 Department of Psychology, University of Wuppertal, Wuppertal, Germany; 4 Department of Psychology, University of Michigan, Ann Arbor; 5 Eisenberg Family Depression Center, University of Michigan, Ann Arbor

## Abstract

**Importance:**

Clinical theory and behavioral studies suggest that people experiencing suicidal crisis are often unable to find constructive solutions or incorporate useful information into their decisions, resulting in premature convergence on suicide and neglect of better alternatives. However, prior studies of suicidal behavior have not formally examined how individuals resolve the tradeoffs between exploiting familiar options and exploring potentially superior alternatives.

**Objective:**

To investigate exploration and exploitation in suicidal behavior from the formal perspective of reinforcement learning.

**Design, Setting, and Participants:**

Two case-control behavioral studies of exploration-exploitation of a large 1-dimensional continuous space and a 21-day prospective ambulatory study of suicidal ideation were conducted between April 2016 and March 2022. Participants were recruited from inpatient psychiatric units, outpatient clinics, and the community in Pittsburgh, Pennsylvania, and underwent laboratory and ambulatory assessments. Adults diagnosed with borderline personality disorder (BPD) and midlife and late-life major depressive disorder (MDD) were included, with each sample including demographically equated groups with a history of high-lethality suicide attempts, low-lethality suicide attempts, individuals with BPD or MDD but no suicide attempts, and control individuals without psychiatric disorders. The MDD sample also included a subgroup with serious suicidal ideation.

**Main Outcomes and Measures:**

Behavioral (model-free and model-derived) indices of exploration and exploitation, suicide attempt lethality (Beck Lethality Scale), and prospectively assessed suicidal ideation.

**Results:**

The BPD group included 171 adults (mean [SD] age, 30.55 [9.13] years; 135 [79%] female). The MDD group included 143 adults (mean [SD] age, 62.03 [6.82] years; 81 [57%] female). Across the BPD (χ^2^_3_ = 50.68; *P* < .001) and MDD (χ^2^_4_ = 36.34; *P* < .001) samples, individuals with high-lethality suicide attempts discovered fewer options than other groups as they were unable to shift away from unrewarded options. In contrast, those with low-lethality attempts were prone to excessive behavioral shifts after rewarded and unrewarded actions. No differences were seen in strategic early exploration or in exploitation. Among 84 participants with BPD in the ambulatory study, 56 reported suicidal ideation. Underexploration also predicted incident suicidal ideation (χ^2^_1_ = 30.16; *P* < .001), validating the case-control results prospectively. The findings were robust to confounds, including medication exposure, affective state, and behavioral heterogeneity.

**Conclusions and Relevance:**

The findings suggest that narrow exploration and inability to abandon inferior options are associated with serious suicidal behavior and chronic suicidal thoughts. By contrast, individuals in this study who engaged in low-lethality suicidal behavior displayed a low threshold for taking potentially disadvantageous actions.

## Key Points

**Question:**

Is the inability to explore multiple alternatives and take advantage of the best options associated with suicidal behavior?

**Findings:**

In 2 case-control studies of adults with borderline personality disorder and depression, inability to fully explore available options was associated with medically serious suicide attempts. In an ambulatory study, this pattern predicted suicidal ideation.

**Meaning:**

The findings suggest that the inability to explore a full range of solutions in a state of suicidal crisis may prevent one from discovering alternatives to attempting suicide; exploring novel ways to cope may help individuals build their safety plans.

## Teaser

This case-control study explores the exploration-exploitation dilemma in suicidal behavior.

## Introduction

People who survive suicide attempts usually come to regret their choice of suicide attempt over constructive alternatives,^1^ suggesting that this choice is often an error of decision-making. Individuals who use substances or gamble in real life^2,3^ and do not make optimal value-based choices or effectively learn from rewards and punishments in the laboratory^4,5,6,7,8,9,10,11,12^ may be more vulnerable to suicidal behavior. However, our understanding of decision-making in a state of suicidal crisis is limited by the reliance on simple decision tasks used in case-control studies that leave out critical real-life demands. In a crisis, decisions are often made during a complex sensorimotor interaction, as one may take a phone call, read an upsetting message, walk, look around, and even begin to implement a suicidal plan. There is usually real or perceived time pressure, and a vast number of options may become available and vanish dynamically. Imagine a person experiencing unbearable distress who may consider drinking alcohol, taking an overdose, going for a walk while practicing a coping skill, or calling a friend. Drinking and overdose are always there, while the availability and worth of alternatives can change: the friend may not answer the call after a work shift starts, and a walk may bring no relief once the afternoon heat sets in. Many alternatives may remain, but it is hard to consider which ones would work when experiencing a sense of crisis.

Clinical theories describe the suicidal crisis as a myopic, passive, and constricted cognitive state—one of tunnel vision.^13,14,15^ While clinical accounts yield few predictions about neurobehavioral mechanisms, reinforcement learning^16,17,18,19^ provides a useful theoretical framework for understanding decision-making. Dynamic decision-making involves a continuous competition between available actions,^20,21,22^ and adaptive behavior depends on resolving this competition.^23^ Reinforcement learning frames option competition as a dilemma between exploiting options thought to be best and exploring potentially superior alternatives.^24^ In this explore-exploit framework, we can view cognitive constriction as a narrow and ineffective exploration, yielding a subset of suboptimal choices. Returning to our example, one may end up drinking alcohol or even attempting suicide, having not explored constructive solutions when they were available and useful.

**Figure 1.**
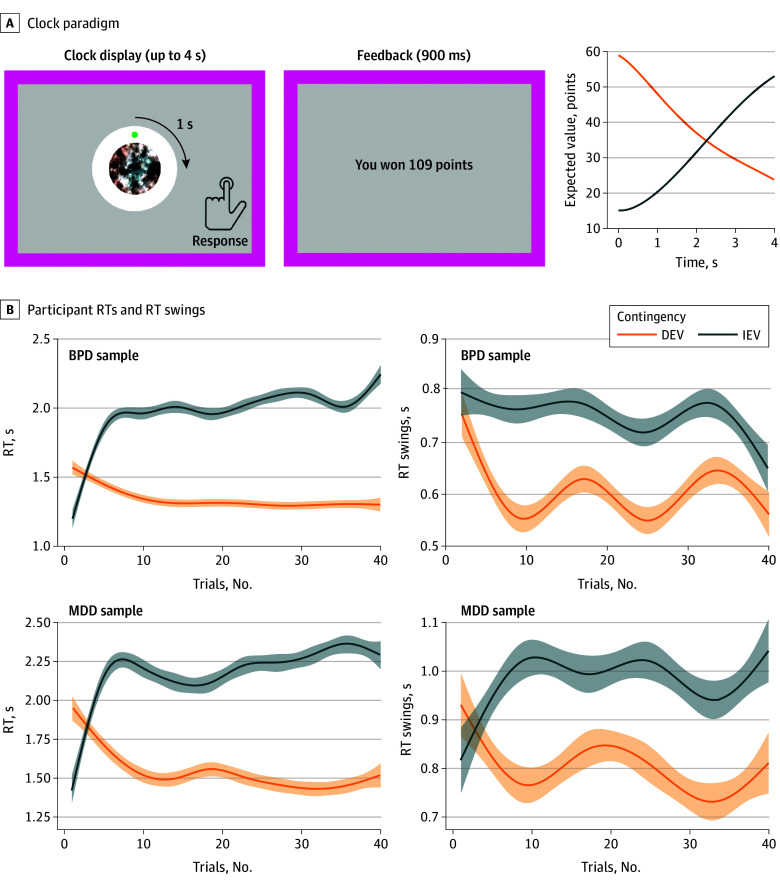
Clock Task and Behavioral Manipulation Checks A, The clock paradigm consists of decision and feedback phases. During the decision phase, a dot revolves 360° around a central stimulus over the course of 4 seconds. Participants press a button to stop the revolution and receive a probabilistic outcome. During the feedback phase, participants are informed about the number of points they won on this trial, with 0 points representing reward omission. Rewards are drawn from 1 of 2 monotonically time-varying contingencies in which expected values of choices either increase (increasing expected value [IEV]) or decrease (decreasing expected value [DEV]) with prolonged wait. Reward probabilities and magnitude varied independently (eFigure 1 in Supplement 1). B, Evolution of participants’ response times (RT) and RT swings by contingency in the borderline personality disorder (BPD) and major depressive disorder (MDD) samples. Plotted data are smoothed using a generalized additive model (GAM) in the *ggplot2* package of R version 3.4.4 (R Foundation). In subplots on the right, the smoothing used natural splines from the *splines* package in R version 4.3.2, with a basis of 5 knots. The shaded area around the lines represents 95% CIs. Participants learned to respond later in the IEV compared to the DEV condition and RT swings generally decreased later in learning (especially in IEV). To ascertain that time courses were not distorted by smoothing, trial-averaged data are presented in eFigures 2 and 3 in Supplement 1. The difference between DEV and IEV at trial 1 is due to the alternation of IEV and DEV conditions, which change every 40 trials of the task.

To understand how people who are vulnerable to suicide resolve option competition under time pressure, we investigated the exploration-exploitation of a continuous space where a large number of options become available and vanish dynamically. We used the clock task^25^ (Figure 1A), where movement through a 1-dimensional environment is signaled by a dot rotating around a circle and rewards, and the distance between consecutive choices provides a straightforward measure of exploration. To assess exploitation, we used a previously validated computational model that explores and exploits efficiently in a resource-rational manner.^26^ During learning, when one chooses among discrete options, it is hard to infer that a given choice is exploratory without computational modeling. By contrast, shifts to far-away locations of a continuous space, particularly when unexplained by reward history, are likely to reflect exploration. Much exploration on the clock task results from shifting away immediately after unrewarded responses. Humans and other mammals consistently display these so-called win-stay/lose-shift responses alongside reinforcement learning.^27,28,29,30^ Critically, smaller win-stay/lose-shift responses were associated with attempted suicide in our earlier armed bandit studies of late-life depression.^8^ Here, we aimed to understand whether this impairment—and ostensibly the inability to find solutions in a suicidal crisis—may reflect deficits in exploration (including by shifting far away from unrewarded options) or in exploitation based on longer-term learned values.^4,5,6,7,8,9,10,11,12^

Surveying diverse forms of suicidal behavior maximally representative of death by suicide, we examined exploration-exploitation in people with borderline personality disorder (BPD) and late-life depression who had made high-lethality vs low-lethality suicide attempts. Whereas BPD is characterized by affective instability, rash decisions, and recurrent suicidal thoughts and behaviors,^31,32^ suicidal acts in individuals with late-life depression are less frequent but more determined and lethal.^33,34,35^ Finally, to validate our case-control findings prospectively, we examined whether behavioral exploration/exploitation predicted incident suicidal thoughts assessed via ecological momentary assessment. We hypothesized that high-lethality suicide attempts and incident suicidal thoughts would be associated with underexploration, particularly following unrewarded choices, and with inability to exploit.

## Methods

### Participants

Participants (Table 1; eTables 1 and 2 in Supplement 1) included 171 adults with BPD and 143 adults with major depressive disorder (MDD). We contrasted individuals with high-lethality suicide attempts with those with low-lethality attempts, patients with no history of suicide attempts, and psychiatrically healthy control individuals. To identify deficits specific to attempted rather than merely contemplated suicide, we included a group with suicidal ideation with a plan but no attempt history in the MDD sample only. See the eMethods in Supplement 1 for full clinical and psychological characterization of the samples. This study followed the Enhancing the Quality and Transparency of Health Research (EQUATOR) reporting guideline. The institutional review board of the University of Pittsburgh approved the study procedures. Written informed consent was obtained before participation.

**Table 1.** Case-Control Study Groups Across Samples

Sample	Group	Age, mean (SD), y	Sex, No (%)		Participant count
			Female	Male	
BPD (age range at enrollment: 18-45 y)^a^	Healthy control	30.5 (9.0)	40 (74.1)	14 (25.9)	54
	BPD with no prior history of suicidal behavior	29.3 (6.5)	24 (75.0)	8 (25.0)	32
	BPD with low-lethality suicide attempt	29.6 (8.8)	39 (79.6)	10 (20.4)	49
	BPD with high-lethality suicide attempt	33.1 (11.4)	32 (88.9)	4 (11.1)	36
MDD (age range at enrollment: 50-80 y)	Healthy control	63.3 (8.2)	23 (53.5)	20 (46.5)	43
	MDD with no self-injurious behavior or ideation	61.9 (6.7)	15 (46.9)	17 (53.1)	32
	MDD with suicidal ideation and plan	61.5 (5.0)	18 (58.1)	13 (41.9)	31
	MDD with low-lethality suicide attempt	61.8 (7.1)	17 (70.8)	7 (29.2)	24
	MDD with high-lethality suicide attempt	59.8 (5.2)	8 (61.5)	5 (38.5)	13

Abbreviations: BPD, borderline personality disorder; MDD, major depressive disorder.

^a^ A subset of 119 participants in the BPD group also completed a 21-day ecological momentary assessment (EMA) protocol assessing daily instances of suicidal ideation. Due to low daily levels of suicidal ideation in the healthy control group, the EMA portion of analyses focused exclusively on 84 individuals with BPD (26 with no suicide attempts, 40 with low-lethality attempts, and 18 with high-lethality attempts). The medical seriousness of attempts was assessed using the Beck Lethality Scale (BLS). For individuals with multiple suicide attempts, data for the highest lethality attempt were used. High-lethality suicidal behavior was defined as a BLS score of 4 or greater.

### Daily Assessments

Participants completed a 21-day ecological momentary assessment protocol (6 surveys per day) within predefined time windows. Suicidal ideation was assessed with 2 dichotomous items (1 = yes, 0 = no^36,37^) from the Columbia Suicide Severity Rating Scale^38^: “Have you wished you were dead or wished you could go to sleep and not wake up?” and “Have you actually had any thoughts of killing yourself?” We averaged across the instances of endorsements of suicidal ideation over the duration of the ecological momentary assessment protocol to get an index of frequency.

### Clock Task

All participants explored and exploited a 1-dimensional continuous space on the clock task^25^ (Figure 1; eMethods and eFigures 1-4 in Supplement 1) over the course of 240 trials. During the decision phase, a green dot revolved 360° around a central stimulus. Participants were informed that the timing of their response controlled the number of points they could win and that not responding during a single revolution (4 seconds in the BPD sample and 5 seconds in the MDD sample) would leave them with no points on that trial. They pressed a button to stop the revolution and received probabilistic feedback controlled by 2 difficult contingencies, such that expected values of choices either increased or decreased along the interval. Reward probabilities and magnitude varied independently. Contingencies reversed every 40 trials and, to rule out the effects of novelty on task behavior, the MDD participants were not signaled about these changes.

### Computational Modeling

**Figure 2.**
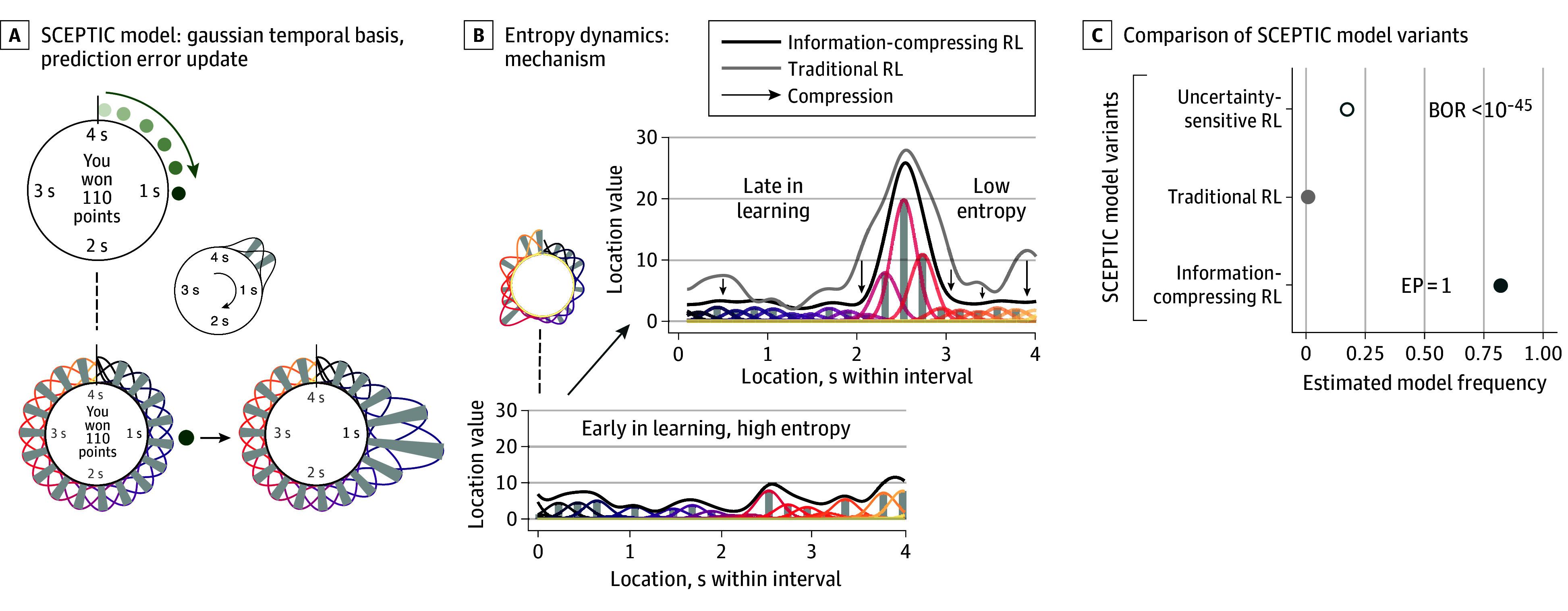
Model Description, Selection, and Comparison A, The Strategic Exploration/Exploitation of Temporal Instrumental Contingencies (SCEPTIC) reinforcement learning model shows basis function representation. Top: participant responds at 1 second and wins 110 points. Bottom left: the 1-dimensional space of the task is tiled with Gaussian-shaped learning elements with staggered receptive fields. Bottom right: the reward at 1 second updates expected values (weights) of nearby basis elements. Color indicates the location of the basis function within the interval. Darker colors indicate earlier responses, and lighter colors indicate later responses. B, Entropy dynamics of the information-compressing reinforcement learning (RL) model. Early in learning, entropy is high because all locations have similar values. The figure shows example value distribution early in learning, first within the circular visual space of the task and then projected linearly onto the abscissa. Later in learning, entropy decreased as the most attractive option dominated. Traditional RL is contrasted with information-compressing RL. Information compression (arrows) reduces the entropy of the value distribution. In contrast to traditional RL with long-term value persistence, information-compressing RL learns and forgets faster. Information compression is an emerging property of the algorithm, resulting from both the decay of unchosen options and value updates of the chosen location. In contrast, entropy change in the traditional RL model depends only on the latter. Entropy was defined as Shannon entropy of the normalized vector of element weights (gray bars). C, Random-effects bayesian model comparison of SCEPTIC model variants. Dots represent the estimated model frequency (ie, the proportion of participants for whom a given model provided the best fit to the data). The shade matches that of the same models in panel B. Uncertainty-sensitive RL is the SCEPTIC model variant where choice was influenced by both uncertainty and reward value to embody the alternative hypothesis that uncertainty modulates exploration. Information-compressing RL was used in the study as it performed better than traditional or uncertainty-sensitive RL. Diagnostic groups exhibited a similar pattern of model fits to the full sample. BOR indicates bayesian omnibus risk; EP, exceedance probability.

Computational modeling is illustrated in Figure 2 and the eMethods, eTable 3, and eFigure 5 in Supplement 1. Our goal was to identify the highest-value region of the space, which a successful agent would be exploiting on any given trial, given each participant’s sampling and reinforcement history using reinforcement learning. Thus, we fitted our previously validated (across environments and levels of analysis^26,39,40^) Strategic Exploration/Exploitation of Temporal Instrumental Contingencies (SCEPTIC) model to participants’ choices. SCEPTIC reduces the potentially infinite continuous options to a handful of discrete actions, using learning elements with staggered receptive fields implemented as gaussian temporal basis functions.^39^ To explore and exploit efficiently, while reducing memory load, SCEPTIC selectively maintains the values of preferred actions and allows the nonpreferred alternatives to decay. To improve precision, model parameters were estimated by an empirical bayesian procedure using the variational bayesian approach,^41^ regularizing individual estimates by the group posterior.

### Data Analysis

Measures of exploration and exploitation are detailed in Table 2 and the eMethods in Supplement 1. As in our previous studies,^26,39^ we examined individual differences in exploration and exploitation in multilevel linear regression models predicting trial-by-trial response times (RTs) implemented in the lme4 package of R version 1.1.35.1 (R Foundation), accounting for random intercepts for each participant and run and, in sensitivity analyses, participant-level random slopes of behavioral variables. Missed responses and RTs less than 200 ms were excluded (BPD sample: 341 of 41 040 trials [0.83%]; MDD sample: 517 of 34 320 trials [1.51%]).

**Table 2.** Definitions

Concept	General definition	Operationalization: clock task and SCEPTIC model
Exploration	Sampling a broader range of options to find the ones with highest value.	RT swings represent the distance between consecutive responses and thus provide a model-free index of exploration. To reduce the confounds of sensorimotor precision, this is estimated as the effect of RT in the previous trial on RT in the current trial in multilevel models of behavior. Weaker RT autocorrelation reflects greater RT swings. As detailed in the eMethods in Supplement 1, RT swings mostly reflect random rather than strategic exploration.
Exploitation	Choosing options currently thought to yield the highest rewards.	The RT(Vmax) is the location of the best option, the response time with the highest expected value, or the global value maximum. RT(Vmax) is the best response location according to the SCEPTIC model, given what options the participant has sampled so far and the rewards received. By choosing RT(Vmax), one maximizes their short-term reward. Therefore, effect of RT(Vmax) on choice indexes the rate of exploitation.
Entropy of the value function: global uncertainty	Value function is the expected reward (value) associated with each option that is being tracked by a learning agent. Its entropy (information content) scales with the number of competing options and tunes the explore-exploit balance. In other words, entropy reflects uncertainty as to which option yields the highest expected reward. Entropy is maximal when all options appear equally attractive, which is often the case when their true value is unknown. Therefore, high entropy promotes exploration and hence discovery of better options. For example, at a food market in an exotic city, a person may start with no idea which food is best (high entropy: many seemingly good options). They may then randomly sample (explore) different foods, but as they start liking certain foods more (low entropy: several best options dominate), they will focus on (exploit) those options more.	SCEPTIC approximates the value (expected reward) at each location on the clock with a set of learning elements whose temporal receptive fields cover the time interval. Each element updates its weight through the discrepancy between model-predicted reward at the chosen RT and the temporally proximal obtained reward. Shannon entropy (information content) of the normalized vector of element weights (values) is high earlier in learning in the presence of multiple competing options and decreases when a single most attractive option begins to dominate.

Abbreviations: RT, response time; SCEPTIC, Strategic Exploration/Exploitation of Temporal Instrumental Contingencies.

Participants’ tendency to explore was measured by the decreased effect of RT in the previous trial (RT[t-1]) on RT in the current trial (RT[t]), conceptually corresponding to the tendency to alternate between early and late parts of the interval (RT swings) and primarily reflecting random rather than uncertainty-directed exploration.^39^ Its interaction with reward quantified the tendency to shift away from unrewarded choices, while the interaction with trial specifically tested adaptive early exploration. These large RT swings of 1 to 2 seconds far exceed the threshold of sensorimotor precision. Participants’ ability to exploit was captured by the effect of RT with the highest expected value, as predicted by the SCEPTIC model (RT[Vmax]), on their choices. The RT(Vmax) × trial interaction tested the transition from earlier exploration to later exploitation.

## Results

### Exploration in the BPD Sample

**Figure 3.**
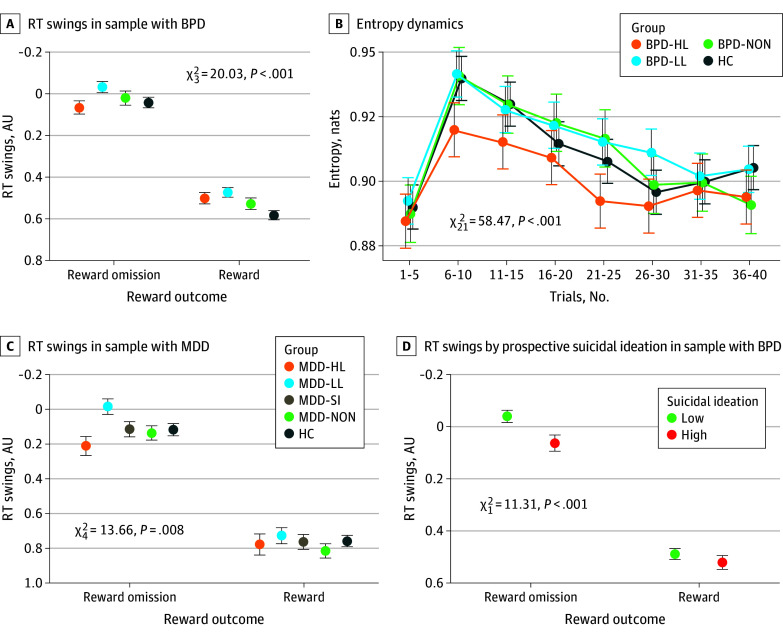
Behavior on the Clock Task Estimates from multilevel linear regression models predicting trial-level responses. See eTables 4, 18, 19, and 22 in Supplement 1, respectively, for full outputs of these models. A, Response time (RT) swings following reward vs omission by group in the borderline personality disorder (BPD) sample. Smaller numbers indicate larger RT swings and vice versa. Individuals with high-lethality (HL) suicide attempts had lower levels of win-stay/lose-shift behavior (ie, the tendency to repeat rewarded options and shift away from unrewarded options). Whereas individuals with HL suicide attempts were less likely to shift behavior after a previously unrewarded action (smaller lose-shift), individuals with low-lethality (LL) suicide attempts were less likely to stick with the previously rewarded actions (smaller win-stay). B, Entropy dynamics by group during the clock task in the BPD sample. The ordinate depicts Shannon entropy of normalized element weights (illustrated in Figure 2), with higher values reflecting a greater number of competing options. Individuals with HL suicide attempts discovered fewer options than other groups, as evidenced by a lack of value entropy expansion in that group. C, Response time swings following reward vs omission as in panel A, replication in the major depressive disorder (MDD) sample. D, Response time swings following reward vs omission on the clock task and prospective suicidal ideation during ecological momentary assessment in individuals with BPD. Lower levels of win-stay/lose-shift (especially after reward omission) were associated with more frequent suicidal ideation in daily life. AU indicates arbitrary units; HC, healthy control individuals; NON, individuals without lifetime history of suicide attempts; SI, suicidal ideation.

Results for exploration in the BPD sample (mean [SD] age, 30.55 [9.13] years; 135 [79%] female and 36 [21%] male) are shown in Figure 3A and eTable 4 in Supplement 1. Levels of exploration differed across groups (group × RT[t-1]: χ^2^_3_ = 50.68; *P* < .001). Follow-up analyses revealed smaller RT swings (lower exploration) in individuals with BPD and high-lethality suicide attempts vs those with BPD and low-lethality suicide attempts (*t*_39,350_ = −4.32; *P* < .001) even after accounting for the effects of reward described below. Further, both individuals with BPD and low-lethality attempts (*t*_39,340_ = −7.01; *P* < .001) and those with BPD and no suicide attempts (*t*_39,310_ = −2.63; *P* = .008) had larger RT swings compared to control individuals. Thus, we observed a striking heterogeneity among individuals with suicidal behavior, with relatively low exploration in individuals with BPD and high-lethality suicide attempts and relatively high exploration in those with BPD and low-lethality suicide attempts. We found no selective impairment in early exploration above and beyond these differences.

Much exploration on the clock task depends on RT swings away from unrewarded choices (lose-shifts).^39^ Our analysis found a group × reward × RT[t-1] interaction (χ^2^_3_ = 20.03; *P* < .001). Follow-up analyses revealed diminished win-stay/lose-shift responses in individuals with BPD and high-lethality suicide attempts vs all other groups (BPD with low-lethality attempts: *t*_38,950_ = 2.81; *P* = .004; those with BPD and no suicide attempts: *t*_39,020_ = 2.64; *P* = .008; control individuals: *t*_38,960_ = 4.46; *P* < .001). Qualitatively, while all BPD groups, particularly those with low-lethality attempts, displayed smaller win-stays; those with BPD and high-lethality attempts displayed smaller lose-shifts, particularly vs those with BPD and low-lethality attempts, but also vs those with BPD and no suicide attempts. Group differences persisted after controlling for the levels of depressive symptoms, suicide attempt recency (although recency predicted smaller lose-shifts), impulsivity, medication exposure, estimated premorbid IQ, and executive function (eTables 5-13 in Supplement 1). Smaller lose-shifts among individuals with BPD and high-lethality attempts could also be due to a working memory deficit; however, this alternative explanation was also ruled out (eTable 14 in Supplement 1). Group differences were not explained by individual heterogeneity of behavioral effects, as indicated by sensitivity analyses including random slopes of behavioral variables (eTables 15-17 in Supplement 1), or by suppressor effects (eTable 18 in Supplement 1).

To ascertain whether individuals with BPD and high-lethality suicide attempts indeed underexplored the option space, we used the SCEPTIC model to examine information dynamics reflective of option competition, finding that individuals with BPD and high-lethality attempts discovered fewer options than other groups (Figure 3B; eTable 19 in Supplement 1).

### Replication: Exploration in the MDD Sample

Results for exploration in the MDD sample (mean [SD] age, 62.03 [6.82] years; 81 [57%] female and 62 [43%] male) are shown in Figure 3C and eTable 20 in Supplement 1. Levels of exploration differed across groups (group × RT[t-1]: χ^2^_4_ = 36.34; *P* < .001). As in the BPD sample, follow-up analyses revealed smaller RT swings in individuals with MDD and high-lethality suicide attempts vs those with MDD and low-lethality suicide attempts (*t*_33,170_ = −5.12; *P* < .001), as well as vs those with MDD and suicidal ideation but no attempt (*t*_33,170_ = −2.13; *P* = .03) and control individuals (*t*_33,170_ = −2.20; *P* = .03). We found no group differences in early exploration specifically.

As in the BPD sample, group differences in exploration were further qualified by reward (group × reward × RT[t-1]: χ^2^_4_ = 13.66; *P* = .008). Individuals with MDD and high-lethality suicide attempts had diminished win-stay/lose-shift responses compared to individuals with MDD and no suicide attempts (*t*_33,160_ = 2.33; *P* = .02) and those with MDD and low-lethality suicide attempts (*t*_33,170_ = 3.48; *P* < .001). Again, relative to other groups, whereas individuals with MDD and high-lethality suicide attempts displayed smaller lose-shifts, those with MDD and low-lethality attempts exhibited greater lose-shifts.

### Exploitation in the BPD Sample

Results for exploitation in the BPD sample are shown in eTable 4 in Supplement 1. After accounting for the effects of the last reward described above, omnibus tests showed no overall group differences in exploitation; however, individuals with BPD and high-lethality suicide attempts displayed higher levels of exploitation vs control individuals (*t*_34,980_ = −2.31; *P* = .02) but not vs other groups (|*t*| ≤ 1.16). Since partialing out the effects of last reward (reward × RT[t-1]) from effects of long-term reinforcement (RT[Vmax]) constitutes overcontrolling, we tested a model (eTable 21 in Supplement 1) omitting the reward × RT[t-1] term, finding that levels of overall exploitation differed across groups (group × RT[Vmax]: χ^2^_3_ = 9.20; *P* = .03): individuals with BPD and high-lethality suicide attempts exhibited higher exploitation vs control individuals (*t*_36,510_ = −2.70; *P* = .007) but not vs other groups.

### Replication: Exploitation in the MDD Sample

Results for exploitation in the MDD sample are shown in eTable 20 in Supplement 1. We found no group differences in exploitation.

### Exploration, Exploitation, and Prospectively Assessed Suicidal Thoughts

Results pertaining to suicidal thoughts are illustrated in Figure 3D and eTable 22 in Supplement 1. Eighty-four individuals with BPD completed the 21-day ecological momentary assessment study, with 56 of these reporting suicidal thoughts (present on average 7% of days; median [range], 2% [0%-94%]), enabling us to examine the associations between exploration-exploitation on the clock task and prospectively assessed suicidal thinking in daily life.

Prospective suicidal ideation was associated with the same pattern of lower exploration (χ^2^_1_ = 30.16; *P* < .001) and smaller lose-shifts (χ^2^_1_ = 11.31; *P* = .001) as individuals with high-lethality suicide attempts (Figure 3A, B, and C). No selective association was observed with early vs late exploration. Results remained qualitatively unchanged when excluding 2 participants with extreme frequencies of suicidal ideation (40% and 94% days) or controlling for suicide attempt recency or affective predictors of suicidal ideation (negative internalizing, externalizing, and impulsive affect during ecological momentary assessment) (eTables 23-27 in Supplement 1).

## Discussion

The behavioral experiments in this case-control study, augmented with reinforcement-learning modeling, found associations between serious suicidal behavior in both borderline personality disorder and late-life depression and an inability to shift away from unrewarded choices resulting in the underexploration of a continuous option space. This narrow, inflexible behavior prospectively predicted daily suicidal ideation. By contrast, low-lethality suicidal behavior in both individuals with BPD and depression was associated with excessive shifts after rewarded as well as unrewarded actions. These associations were not explained by plausible confounds, including medication exposure, depressive symptoms, premorbid IQ, executive function, behavioral heterogeneity, and affective predictors of suicidal ideation.

Earlier studies using armed bandits found associations between high-lethality suicidal behavior in mid- and late-life depression and deficits in learning and behavioral adaptation.^8,42^ Supporting these associations, the present findings reveal that, given a choice among many uncertain options under time pressure, individuals at the highest risk explore only a limited subset, sticking with unrewarded choices. To our knowledge, this behavioral pattern has not been described in psychopathology research; it diverges from the performance of patients with schizophrenia, for example, on the same task.^43^ It is equally distinct from win-shift behavior on bandit tasks we previously observed in individuals who attempted suicide,^8^ potentially indicating multiple deficits with additive or similar effects on suicide risk. What neurocomputational deficits may underlie an inability to shift away from unrewarded choices? In rodents, lose-shift behavior depends on the lateral striatum,^29,44^ a sensorimotor region approximately homologous to the primate dorsolateral putamen. In contrast, win-stay rodent behavior depends on the ventromedial striatum^44^ and lateral habenula.^45^ Interestingly, however, human lose-shift responding increases under cognitive load, suggesting that frontoparietal control may suppress automatic, striatum-mediated lose-shifts.^46^ During exploration and learning in continuous spaces, dynamic maps of competing options are found in frontoparietal circuits, specifically the dorsal stream and caudal posterior parietal cortex .^40,47^ One intriguing possibility is that inappropriately rigid or exaggerated frontoparietal responses to option competition suppress adaptive lose-shift behavior in people who are prone to serious suicidal behavior. Conversely, excessive lose-shifts in individuals with low-lethality suicide attempts may be related to a disrupted encoding of the longer-term reinforcement history we previously described in attempted suicide.^4,7^

Our observations resonate with clinical notions of cognitive constriction and tunnel vision and provide a fine-grained behavioral and computational account of the suicide diathesis. Curiously, although the present study was not designed to distinguish between traitlike and statelike deficits, exploratory analyses of suicide attempt recency (eTables 5 and 22 in Supplement 1) suggest that decreased lose-shift responding may be a state-modulated trait. At the same time, our results highlight the role of trait impairments in decision capacity, which can facilitate serious suicidal behavior in a crisis, consistent with the view of suicide as an unintentional decision where the demands of a crisis exceeded one’s decision-making capacity.^16,48^ Specifically, individuals prone to underexploration are more likely to select an often-used (or considered) solution in a crisis in lieu of adaptively exploring potentially better alternatives. In psychotherapy, exploring solutions one had never tried before may be a useful skill to learn and practice both at an emotional baseline and when distressed.

It has been questioned whether phenomena such as passive death wish, suicidal thoughts, and more vs less medically serious suicidal acts belong to a single severity continuum and whether underlying risk factors differ only quantitatively or also qualitatively.^49,50^ Consistent behavioral differences between individuals with high-lethality and low-lethality suicide attempts controvert the continuum model, pointing instead to qualitatively distinct behavioral pathways. One is generally skeptical of studies where the performance of distinct clinical groups falls on both sides of healthy control individuals, since this pattern often reflects merely unexplained interindividual heterogeneity. However, here, the behavioral divergence between individuals with high-lethality vs low-lethality suicide attempts was replicated across different clinical populations and was robust to statistical controls for individual heterogeneity and plausible confounds. Furthermore, the behavioral distinctiveness of high-lethality suicide attempts from other forms of suicidal behavior and ideation has been observed in several previous studies across clinical populations and samples,^4,5,7,8,11^ suggesting that this divergence is systematic. Thus, it is likely that qualitatively distinct behavioral pathways lead to high-lethality suicide attempts and, by extension, many suicide deaths vs lower-lethality suicide attempts. While the first pathway is marked by narrow and inflexible choices, the second is characterized by excessive behavioral plasticity in response to failures, which may correspond to a lower threshold for engaging in potentially disadvantageous and specifically suicidal behavior. Additionally, maximum attempt lethality—a hard outcome (relative to one’s level of intent or planning)—must be considered a key dimension of past suicidal behavior in both research and practice.

Contrary to expectations, we found no evidence that people prone to suicide are unable to exploit the best of previously sampled options, with the caveat that the individuals at the highest risk were choosing from a more limited set than other participants. If anything, there was some evidence of overexploitation in the borderline personality disorder sample, with no group differences in the depression sample. Considering prior evidence associating suicidal behavior with disadvantageous value-based choices,^5,11,51^ our findings suggest that such behavior may instead reflect an admixture of overly rigid and erratic behavioral patterns, challenging the notion of simple insensitivity to long-term value.

### Limitations

The case-control design of our study limits causal inferences, a limitation partly offset by prospective validation. Future studies will also need to differentiate strategic from stochastic exploration and examine how affective states shape the set of options under consideration, particularly following adverse outcomes, and test formal accounts of affective meta-reasoning during learning and decision-making.^16,52^

## Conclusions

In summary, divergent behavioral signatures of high-lethality vs low-lethality suicide attempts likely expose distinct neurocognitive pathways. This underscores the need for a broader taxonomy of clinically relevant individual differences in human decision-making.

## Notes

Supplement 1. eMethods eTable 1. Demographic and clinical characterization of the BPD sample eTable 2. Demographic and clinical characterization of the MDD sample eTable 3. Model fits by diagnostic groups eTable 4. BPD sample: Exploration and exploitation on the Clock task eTable 5. BPD sample sensitivity analysis: Levels of depressive symptoms (excluding healthy controls) eTable 6. BPD sample sensitivity analysis: Suicide attempt recency eTable 7. BPD sample sensitivity analysis: Impulsivity eTable 8. BPD sample sensitivity analysis: Medication exposure to antidepressants (excluding healthy controls) eTable 9. BPD sample sensitivity analysis: Medication exposure to opioids (excluding healthy controls) eTable 10. BPD sample sensitivity analysis: Medication exposure to sedatives or hypnotics (excluding healthy controls) eTable 11. BPD sample sensitivity analysis: Medication exposure to antipsychotics (excluding healthy controls) eTable 12. BPD sample sensitivity analysis: Estimated premorbid IQ eTable 13. BPD sample sensitivity analysis: Executive function eTable 14. BPD sample sensitivity analysis: Effects of working memory eTable 15. BPD sample sensitivity analysis controlling for individual random slopes of RT lag eTable 16. BPD sample sensitivity analysis controlling for individual random slopes of RT vmax eTable 17. BPD sample sensitivity analysis controlling for individual random slopes of reward (reward lag) eTable 18. BPD sample: model without RT vmax:Group:trial interaction as a covariate eTable 19. BPD sample: Information dynamics reflective of option competition eTable 20. MDD sample: Exploration and exploitation on the Clock task eTable 21. BPD sample: model without reward lag:RT lag interaction as a covariate eTable 22. BPD EMA subsample: Exploration and exploitation on the Clock task predicting the frequency of prospective daily suicidal ideation eTable 23. BPD EMA subsample sensitivity analysis: Excluding 2 relatively extreme values on daily suicidal ideation measure eTable 24. BPD EMA subsample sensitivity analysis: SA recency eTable 25. BPD EMA subsample sensitivity analysis: Controlling for average levels of negative internalizing affect in daily life eTable 26. BPD EMA subsample sensitivity analysis: Controlling for average levels of externalizing affect in daily life eTable 27. BPD EMA subsample sensitivity analysis: Controlling for average levels of impulsive affect in daily life eFigure 1. Reward magnitude and probability across time-varying contingencies eFigure 2. Behavioral manipulation checks by group (generalized additive model) eFigure 3. Behavioral manipulation checks (raw data, trial averages across groups) eFigure 4. Behavioral manipulation checks (raw data, trial averages by group) eFigure 5. Posterior predictive checks

Supplement 2. Data sharing statement
