# Supplementary material for: Exploration-Exploitation and Suicidal Behavior in Borderline Personality Disorder and Depression
Source: JAMA Psychiatry. 2024 Jul 10;81(10):1010–9. doi: 10.1001/jamapsychiatry.2024.1796 (PMC11238070; doi:10.1001/jamapsychiatry.2024.1796)
Supplement: Supplement 1. — eMethods eTable 1. Demographic and clinical characterization of the BPD sample eTable 2. Demographic and clinical characterization of the MDD sample eTable 3. Model fits by diagnostic groups eTable 4. BPD sample: Exploration and exploitation on the Clock task eTable 5. BPD sample sensitivity analysis: Levels of depressive symptoms (excluding healthy controls) eTable 6. BPD sample sensitivity analysis: Suicide attempt recency eTable 7. BPD sample sensitivity analysis: Impulsivity eTable 8. BPD sample sensitivity analysis: Medication exposure to antidepressants (excluding healthy controls) eTable 9. BPD sample sensitivity analysis: Medication exposure to opioids (excluding healthy controls) eTable 10. BPD sample sensitivity analysis: Medication exposure to sedatives or hypnotics (excluding healthy controls) eTable 11. BPD sample sensitivity analysis: Medication exposure to antipsychotics (excluding healthy controls) eTable 12. BPD sample sensitivity analysis: Estimated premorbid IQ eTable 13. BPD sample sensitivity analysis: Executive function eTable 14. BPD sample sensitivity analysis: Effects of working memory eTable 15. BPD sample sensitivity analysis controlling for individual random slopes of RT lag eTable 16. BPD sample sensitivity analysis controlling for individual random slopes of RT vmax eTable 17. BPD sample sensitivity analysis controlling for individual random slopes of reward (reward lag) eTable 18. BPD sample: model without RT vmax:Group:trial interaction as a covariate eTable 19. BPD sample: Information dynamics reflective of option competition eTable 20. MDD sample: Exploration and exploitation on the Clock task eTable 21. BPD sample: model without reward lag:RT lag interaction as a covariate eTable 22. BPD EMA subsample: Exploration and exploitation on the Clock task predicting the frequency of prospective daily suicidal ideation eTable 23. BPD EMA subsample sensitivity analysis: Excluding 2 relatively extreme values on daily suicidal ideation measur [file jamapsychiatry-e241796-s001.pdf]

Supplemental Online Content

Tsypes A, Hallquist MN, Ianni A, Kaurin A, Wright AGC, Dombrovski AY. Exploration-exploitation and suicidal behavior in borderline personality disorder and depression. *JAMA Psychiatry*. Published online July 10, 2024. doi:10.1001/jamapsychiatry.2024.1796

eMethods

eTable 1. Demographic and clinical characterization of the BPD sample

eTable 2. Demographic and clinical characterization of the MDD sample

eTable 3. Model fits by diagnostic groups

eTable 4. BPD sample: Exploration and exploitation on the Clock task

eTable 5. BPD sample sensitivity analysis: Levels of depressive symptoms (excluding healthy controls)

eTable 6. BPD sample sensitivity analysis: Suicide attempt recency

eTable 7. BPD sample sensitivity analysis: Impulsivity

eTable 8. BPD sample sensitivity analysis: Medication exposure to antidepressants (excluding healthy controls)

eTable 9. BPD sample sensitivity analysis: Medication exposure to opioids (excluding healthy controls)

eTable 10. BPD sample sensitivity analysis: Medication exposure to sedatives or hypnotics (excluding healthy controls)

eTable 11. BPD sample sensitivity analysis: Medication exposure to antipsychotics (excluding healthy controls)

eTable 12. BPD sample sensitivity analysis: Estimated premorbid IQ

eTable 13. BPD sample sensitivity analysis: Executive function

eTable 14. BPD sample sensitivity analysis: Effects of working memory

© 2024 Tsypes A et al. *JAMA Psychiatry.*

eTable 15. BPD sample sensitivity analysis controlling for individual random slopes of RT lag

eTable 16. BPD sample sensitivity analysis controlling for individual random slopes of RT vmax

eTable 17. BPD sample sensitivity analysis controlling for individual random slopes of reward (reward lag)

eTable 18. BPD sample: model without RT vmax:Group:trial interaction as a covariate

eTable 19. BPD sample: Information dynamics reflective of option competition

eTable 20. MDD sample: Exploration and exploitation on the Clock task

eTable 21. BPD sample: model without reward lag:RT lag interaction as a covariate

eTable 22. BPD EMA subsample: Exploration and exploitation on the Clock task predicting the frequency of prospective daily suicidal ideation

eTable 23. BPD EMA subsample sensitivity analysis: Excluding 2 relatively extreme values on daily suicidal ideation measure

eTable 24. BPD EMA subsample sensitivity analysis: SA recency

eTable 25. BPD EMA subsample sensitivity analysis: Controlling for average levels of negative internalizing affect in daily life

eTable 26. BPD EMA subsample sensitivity analysis: Controlling for average levels of externalizing affect in daily life

eTable 27. BPD EMA subsample sensitivity analysis: Controlling for average levels of impulsive affect in daily life

eFigure 1. Reward magnitude and probability across time-varying contingencies

eFigure 2. Behavioral manipulation checks by group (generalized additive model)

eFigure 3. Behavioral manipulation checks (raw data, trial averages across groups)

eFigure 4. Behavioral manipulation checks (raw data, trial averages by group)

eFigure 5. Posterior predictive checks

This supplemental material has been provided by the authors to give readers additional information about their work.

eMethods

Sample characteristics (BPD and MDD)

All study procedures were approved by the Institutional Review Board of the University of Pittsburgh (BPD sample: STUDY19050210, MDD sample: STUDY19030288). Written informed consent was obtained prior to study participation. Participants were drawn from longitudinal studies, which recruited from inpatient and outpatient clinics and the Greater Pittsburgh community by advertisement. Participants were required to be between 18 and 45 years of age at the time of enrollment for the BPD sample and between 50 and 80 years at the time of enrollment for the MDD sample. Participants were required not to have a lifetime diagnosis of any psychotic or bipolar disorder, clinical evidence of organic brain disease, physical disorders or treatments with known psychiatric consequences, and an IQ < 70 measured by the Wechsler Test of Adult Reading (WTAR)[1]. To better characterize the sample, we collected demographic information, including participant sex, race, and education levels. Participants reported this information by selecting from a list of options. DSM-IV/5 defined BPD diagnoses were ascertained with the ICD-10-based International Personality Disorder Examination (IPDE[2]). The BPD-NON, MDD-IDE, and MDD-NON groups had no lifetime history of suicide attempts. The BPD-ATT and MDD-ATT groups had a history of self-injurious acts with the intent to die within a 1-month period prior to completing the study assessments or had a history of a past suicide attempt with strong current suicidal ideation at the time of study enrollment. Suicide attempt history was verified by a psychiatrist using all available information: participant's report, medical records, and collateral information from the treatment team, family, and friends. Significant discrepancies between these sources led to exclusion from the study. The medical seriousness of attempts was assessed using the Beck Lethality Scale (BLS[36]). For individuals with multiple suicide attempts, data for the highest lethality attempt was used. High-lethality SB was defined as a BLS score of 4 or greater.

Power analysis

For our main sample (BPD), we aimed to recruit four groups of 30 participants each to achieve 95% power to detect a medium-to-large effect (*f* = .35) using omnibus one-way ANOVA analogous to the LME used in our analyses. This effect size is less than the magnitude of group differences observed in our earlier studies of decision-making (0.44 < *f* < 0.77)[3–5]. Thus, we were well-positioned to detect the effects of interest in our reported analyses with the groups including 32, 36, 49, and 54 participants each.

Assessment of missing values and participants lost to follow-up (main sample: BPD)
355 individuals were examined for eligibility (i.e., were consented to the protocol and pre-screened). Of these individuals, 253 were confirmed eligible through a comprehensive baseline interview. Of these individuals, 179 completed the clock task and 171 passed the data quality checks and were included in our data analyses. Individuals did not pass the data quality checks due to sickness or excessive drowsiness during the task (*n* = 4), random response patterns (*n* = 3), and errors in task administration (*n* = 1).

Experimental paradigm: Clock task[6] (Fig.1)

During the feedback phase, participants were informed about the number of points they had won on this trial. Unbeknownst to the participant, rewards were drawn from one of two monotonically time-varying contingencies: increasing expected value (IEV; waiting longer led to higher rewards) and decreasing expected value (DEV; responding faster on a given trial led to higher rewards). Reward probabilities and magnitude varied independently (eFigure 1). Each trial was followed by an intertrial interval that varied in length based on an exponential distribution. The task was completed in the fMRI scanner and relevant neural analyses will be reported in future manuscripts. Participants across both samples completed a total of 240 trials, with two contingencies reversing every 40 trials. Participants in the BPD sample had to respond within 4s on each trial and were instructed at the beginning of each new contingency that they would once again have to learn to respond at different times to try to win the most points. In contrast, to rule out the effects of novelty on task behavior, participants in the MDD sample were *not* signaled about changes in a contingency at the beginning of each new condition. Further, trials in the MDD sample were extended to 5s to accommodate the slower psychomotor speed in this older sample.

SCEPTIC computational model (schematic depiction: Fig.2)

The SCEPTIC model represents within-trial time (or one-dimensional space) of the clock task using a set of unnormalized Gaussian radial basis functions (**RBFs**) spaced evenly over an interval *T* in which each function has a temporal receptive field with a mean and variance defining its point of maximal sensitivity and the range of times to which it is sensitive, respectively. The basis tracks the expected value of a given choice (response time). To represent **time-varying value**, the heights of each basis function are scaled according to a set of *b* weights, $\mathbf{w} = [w_1, w_2, \ldots, w_b]$.

© 2024 Tsypes A et al. *JAMA Psychiatry.*

The contribution of each basis function to the integrated value depends on its temporal receptive field:

$$\varphi_b(x) = \exp\left[-\frac{(x-\mu_b)^2}{2s_b^2}\right] \quad (1)$$

where *x* is an arbitrary point within the time interval *T*, μ_b is the center (mean) of the RBF, and $s_b^2$is its variance.

More generally, the temporally varying expected value function on a trial *i* is obtained by the multiplication of the weights with the basis:

$$V(i) = \sum_{b=1}^{B} w_b(i)\varphi_b \quad (2)$$

To represent decision-making during the clock task, where the probability and magnitude of rewards varied over the course of each trial, we spaced the centers of 24 Gaussian RBFs evenly across the discrete interval of each trial and chose a fixed width (s_b^2) to represent the temporal variance (width) of each basis function. s_b^2 was chosen such that the distribution of adjacent RBFs overlapped by approximately 50% (see[7] for details and considerations of alternatives).

Traditional RL SCEPTIC model variant (Fig. 2C)

The basic model SCEPTIC variant, referred to as traditional RL in Figure 2C (model comparison), learns the expected values of different response times by updating each basis function *b* according to the equation:

$$w_b(i+1) = w_b(i) + e_b(i|t)\alpha[\text{reward}(i|t) - w_b(i)] \quad (3)$$

where *i* is the current trial in the task, *t* is the observed response time, and *reward* (*i*|*t*) is the reinforcement obtained in trial *i* given the choice *t*. Prediction error updates are weighted by the learning rate α and the temporal generalization function or eligibility e_b. To avoid tracking separate value estimates for each possible moment, feedback obtained at a given response time *t* is propagated to adjacent times. Consequently, to represent temporal generalization of expected value updates, we used a Gaussian RBF centered on the response time t, having width s_g^2. The eligibility e_b of a basis function φ_b to be updated by prediction error is defined as its overlap with the temporal generalization function, *g*:

$$g(x) = \exp\left[-\frac{(x-t)^2}{2s_g^2}\right] \quad (4)$$

$$e_b(i|t) = \int_0^T \min\left[g(\tau), \varphi_b(\tau)\right]d\tau \quad (5)$$

where τ represents an arbitrary timepoint along the interval *T*. Thus, for each RBF *b*, a scalar eligibility e_b between zero and one represents the proportion of overlap between the temporal generalization function and the receptive field of the RBF. In the case of complete overlap, where the response time is perfectly centered on a given basis function, e_b will reach unity, resulting in a maximal weight update according to the learning rule above. Conversely, if there is no overlap between an RBF and the temporal generalization function, e_b will be zero and that RBF will receive no update. Importantly, for the eligibility to be bounded on the interval [0, 1], the basis functions are each normalized to have an area under the curve of unity (i.e., representing probability density). Here, we also fixed the width of the generalization function to match the basis (i.e., $s_g^2 = s_b^2$).

The SCEPTIC model selects an action based on a softmax choice rule, analogous to simpler reinforcement learning problems (e.g., two-armed bandit tasks[8]). For computational speed, we arbitrarily discretized the interval into 100 ms time bins such that the agent selected among 40 potential responses (i.e., a multinomial representation). At trial i the agent chooses a response time in proportion to its expected value:

$$p[t(i+1) = j|V(i)] = \frac{\exp\left(V(i)_j/\beta\right)}{\sum_{\tau=0}^{T}\exp\left(V(i)_\tau/\beta\right)} \quad (6)$$

© 2024 Tsypes A et al. *JAMA Psychiatry.*

where j is a specific response time and the temperature parameter β controls value sensitivity such that choices become more stochastic and less value-sensitive at higher β values.

Uncertainty-sensitive RL SCEPTIC model variant (Fig. 2C)

The SCEPTIC model variant, referred to as the uncertainty-sensitive RL in Figure 2C (model comparison), embodied the alternative hypothesis that local uncertainty modulates exploration. Thus, in this model variant, choice was controlled by a weighted sum of local uncertainty and reward value. Specifically, a fixed learning rate was used for updating the expected value, whereas the posterior uncertainty estimates were updated according to the Kalman gain. The learning rule was:

$$\mu_b(i+1) = \mu_b(i) + e_b(i|t)\alpha[\text{reward}(i|t) - \mu_b(i)] \tag{7}$$

where $\mu_b(i)$ represents the expected value of basis function b on trial i, and α represents the learning rate. The gain for a given basis function, is defined as:

$$k_b(i) = \frac{\sigma_b(i)^2}{\sigma_b(i)^2 + \sigma_{rew}^2} \tag{8}$$

where σ_{rew}^2 represents the expected volatility (measurement noise) of the environment. Here, we provide the model variance of returns from a typical run of the experiment as an initial estimate of measurement noise, although other priors lead to similar model performance. We also initialize prior estimates of uncertainty for each basis function to be equal to the measurement noise, $\sigma_{b0}^2 = \sigma_{rew}^2$, leading to a gain of 0.5 on the first trial (as in ref.[9])

Under the Kalman Filter, uncertainty about expected value for each basis function is represented as the standard deviation of its Gaussian distribution. Likewise, posterior estimates of uncertainty about responses proximate to the basis function b decay in inverse proportion to the gain according to the following update rule:

$$\sigma_b(i+1) = [1 - e_b(i|t)k_b(i)]\sigma_b(i) \tag{9}$$

Estimates of the time-varying value and uncertainty functions are provided by the evaluation of the basis over time:

$$\mathbf{V}(i) = \mu(i)\phi \tag{10}$$

$$\mathbf{U}(i) = \sigma(i)\phi \tag{11}$$

The Fixed U + V policy represents a decision function, Q(i), as a weighted sum of the value and uncertainty functions according to a free parameter, τ. As uncertainty decreases with sampling and expected value increases with learning, value-related information will begin to dominate over uncertainty. Positive values of τ promote uncertainty-directed exploration, whereas negative values yield uncertainty aversion:

$$\boldsymbol{Q}(i) = \mathbf{V}(i) + \tau\mathbf{U}(i) \tag{12}$$

Information-compressing RL SCEPTIC model variant (Fig. 2C)

This SCEPTIC model variant, referred to as the information-compressing RL in Figure 2 (model comparison), was used in the study as it outperformed the traditional and uncertainty-sensitive RL, as well as a number of other plausible alternative models (described in detail in our previous report here[7]). In the information-compressing RL, basis weights corresponding to non-preferred, spatiotemporally distant actions revert toward a prior in inverse proportion to the temporal generalization function:

$$w_b(i+1) = w_b(i) + e_b(i|t)\alpha[\text{reward}(i|t) - w_b(i)] - \gamma\big(1 - e_b(i|t)\big)(w_b(i) - h) \tag{13}$$

where γ is a selective maintenance parameter between zero and one that scales the degree of reversion toward a point h, which is taken to be zero here for parsimony, but could be replaced with an alternative prior expectation. As detailed in our previous report[7], late in learning, selective maintenance compresses the amount of value information represented by the agent by 1/3 to ½ (more in exploitative subjects) and accelerates the transition from exploration to exploitation by accentuating the global value maximum and effacing the values of nonpreferred segments. All of our analyses were based on

© 2024 Tsypes A et al. *JAMA Psychiatry.*

the parameters derived from fitting the information-compressing RL SCEPTIC model to participants' behavior.

We defined the information content of the learned value distribution as Shannon's entropy of the normalized basis weights (the trial index *i* is omitted for simplicity of notation, and tilde denotes normalization):

$$H(\tilde{\mathbf{w}}) = -\sum_{b=1}^{B} \tilde{w}_b \log_{10}(\tilde{w}_b) \tag{14}$$

Weights are normalized here only to calculate entropy, but not within the learning rule (eq. 7, 13). Thus, information compression of the value function is an emergent property of its selective maintenance/decay (eq. 13) and is not merely forced by normalization.

A summary of the features of these and other competing models can be found in Figure 1[7]. Notably, as shown in Supplemental Figure 1[7], parameters for all models in the SCEPTIC fixed learning rate family were recovered with high precision and minimal bias ($R^2 \geq .93$). Further details on model performance across various continuous environments and its ability to account for hippocampal and cortical activity during exploration/exploitation are provided in our previous reports [10,11].

Incorporating SCEPTIC exploitation predictions into frequentist multi-level models alongside the model-free index of exploration

To formally control for person-level confounds, we scale RTs within the condition (IEV vs. DEV), mitigating effects of person-level or even condition-level visuomotor precision while isolating the effect of within-condition trial-varying RT on the next choice, as scaling does not affect autocorrelation. Sensorimotor errors add small-amplitude (relative to the size of the interval) noise to originally intended RTs but cannot introduce, for example, a negative autocorrelation where one alternates between parts of the interval on consecutive trials.

When analyzing RT(t-1) effects as an index of exploration, we control for RT_{Vmax} in the main analyses and rule out multicollinearity as a concern (Variance inflation factors for RT_{Vmax} and RT(t-1) < 3), ensuring that the coefficients for these effects are separately interpretable. Further, we use RT_{Vmax}(t-1) as a predictor alongside the reward(t-1) and the interaction between reward(t-1) and RT(t-1). In this more precise and conservative analysis, RT_{Vmax}(t-1) reflects the longer-term reinforcement history, excluding the last outcome, the effects of which are examined separately. As a result, RT_{Vmax}(t-1) is de-correlated from RT(t-1), reducing multi-collinearity.

Leveraging SCEPTIC value entropy predictions for analyses of exploration

One may still question whether any group differences in exploration apparent in the above analyses are sensitive to assumptions such as interpreting low RT autocorrelation as exploration and partialing out exploitation effects, we aimed to corroborate our main finding in an entirely independent model-based analysis, which instead of RT autocorrelation relies on SCEPTIC-predicted value entropy dynamics. As we illustrate in Figure 2b, as the SCEPTIC agent samples an increasing number of uniquely-valued options early in learning, the Shannon entropy of its normalized value function increases, scaling with the number of competing options. Thus, we expect this expansion of value entropy and, consequently, the number of options explored so far to be attenuated in an agent with a low exploration rate. To test whether high-lethality suicide attempters sample fewer unique options, we entered trial-wise value entropy estimates into a multi-level model with group and trial as predictors, treating individual intercept as random (results reported in Figure 3b).

Data analysis

We used restricted maximum likelihood with the Kenward-Roger degrees of freedom approximation and likelihood ratio chi-square tests for estimating *p* values.

Daily assessments of affect: eTables 24-26

Participants received six random surveys per day assessing suicidal ideation and affect during an approximately 12-hour time window that corresponded to the participants' typical waking hours. Blocked random intervals had a minimum of 90 min between surveys, and participants were given 20 min to initiate a response to each one. In these surveys, participants rated the degree to which they felt nervous, sad, guilty, ashamed, angry, and irritated (i.e., "How ADJECTIVE do you feel right now?"). They also rated the degree to which, since the last prompt, they (1) said or did things they wish they had not; (2) they did something risky; (3) they acted without thinking. Ratings were made on a scale from 1 ("Very slightly or not at all") to 5 ("A great deal"). For the sensitivity analyses, we averaged across the instances of internalizing (mean of nervous, sad, guilty, and ashamed), externalizing (mean of angry and irritated), and impulsive (mean of three three "since the last prompt" items) affect over the duration of the EMA protocol.

© 2024 Tsypes A et al. *JAMA Psychiatry.*

Trait impulsivity: eTable 7

Participants completed the Personality Inventory for Diagnostic and Statistical Manual, Fifth Edition[14]. The disinhibition domain score (i.e., the average of irresponsibility, impulsivity, and distractibility facets) was used in our sensitivity analysis.

References

1. Venegas J, Clark E. Wechsler Test of Adult Reading. In: Kreutzer JS, DeLuca J, Caplan B, eds. *Encyclopedia of Clinical Neuropsychology*. Springer New York; 2011:2693-2694. doi:10.1007/978-0-387-79948-3_1500
2. Loranger AW. International personality disorder examination (IPDE). In: Janca A, Loranger AW, Sartorius N, eds. *Assessment and Diagnosis of Personality Disorders: The ICD-10 International Personality Disorder Examination (IPDE)*. Cambridge University Press; 1997:43-51. doi:10.1017/CBO9780511663215.005
3. Dombrovski AY, Szanto K, Clark L, et al. Corticostriatothalamic Reward Prediction Error Signals and Executive Control in Late-Life Depression. *Psychol Med*. 2015;45(7):1413-1424. doi:10.1017/S0033291714002517
4. Dombrovski AY, Szanto K, Clark L, Reynolds CF, Siegle GJ. Reward signals, attempted suicide, and impulsivity in late-life depression. *JAMA Psychiatry*. 2013;70(10):1020-1030. doi:10.1001/jamapsychiatry.2013.75
5. Vanyukov PM, Szanto K, Hallquist MN, et al. Paralimbic and lateral prefrontal encoding of reward value during intertemporal choice in attempted suicide. *Psychol Med*. Published online 2015:No-Specified. doi:10.1017/S0033291715001890
6. Moustafa AA, Cohen MX, Sherman SJ, Frank MJ. A Role for Dopamine in Temporal Decision Making and Reward Maximization in Parkinsonism. *J Neurosci*. 2008;28(47):12294-12304. doi:10.1523/JNEUROSCI.3116-08.2008
7. Hallquist MN, Dombrovski AY. Selective maintenance of value information helps resolve the exploration/exploitation dilemma. *Cognition*. 2019;183(November 2018):226-243. doi:10.1016/j.cognition.2018.11.004
8. Sutton RS, Barto AG. *Reinforcement Learning: An Introduction*. Second edition. The MIT Press; 2018.
9. Frank MJ, Doll BB, Oas-Terpstra J, Moreno F. Prefrontal and striatal dopaminergic genes predict individual differences in exploration and exploitation. *Nat Neurosci*. 2009;12(8):1062-1068. doi:10.1038/nn.2342
10. Dombrovski AY, Luna B, Hallquist MN. Differential reinforcement encoding along the hippocampal long axis helps resolve the explore–exploit dilemma. *Nat Commun*. 2020;11(1):5407. doi:10.1038/s41467-020-18864-0
11. Hallquist MN, Hwang K, Luna B, Dombrovski AY. Reward-based option competition in human dorsal stream and transition from stochastic exploration to exploitation in continuous space. *Sci Adv*. 2024;10(8):eadj2219. doi:10.1126/sciadv.adj2219
12. Badre D, Doll BB, Long NM, Frank MJ. Rostrolateral prefrontal cortex and individual differences in uncertainty-driven exploration. *Neuron*. 2012;73(3):595-607. doi:10.1016/j.neuron.2011.12.025
13. Fiorillo CD, Newsome WT, Schultz W. The temporal precision of reward prediction in dopamine neurons. *Nat Neurosci*. 2008;11(8):966-973. doi:10.1038/nn.2159
14. Krueger RF, Derringer J, Markon KE, Watson D, Skodol AE. Initial Construction of a Maladaptive Personality Trait Model and Inventory for DSM-5. *Psychol Med*. 2012;42(9):1879-1890. doi:10.1017/S0033291711002674
15. Royall D. Executive Interview. In: Kreutzer JS, DeLuca J, Caplan B, eds. *Encyclopedia of Clinical Neuropsychology*. Springer; 2011:992-997. doi:10.1007/978-0-387-79948-3_1891
16. Hamilton M. The Hamilton Rating Scale for Depression. In: Sartorius N, Ban TA, eds. *Assessment of Depression*. Springer; 1986:143-152. doi:10.1007/978-3-642-70486-4_14
17. Sackeim HA. The definition and meaning of treatment-resistant depression. *J Clin Psychiatry*. 2001;62 Suppl 16:10-17.
18. Parmelee PA, Thuras PD, Katz IR, Lawton MP. Validation of the Cumulative Illness Rating Scale in a Geriatric Residential Population. *J Am Geriatr Soc*. 1995;43(2):130-137. doi:10.1111/j.1532-5415.1995.tb06377.x
19. Beck AT, Steer RA. Manual for the Beck hopelessness scale. *San Antonio TX Psychol Corp*. Published online 1988.

eTable 1. Demographic and clinical characterization of the BPD sample

**Characteristic**	**Nonpsychiatric Controls** (n = 54)	**Borderline Personality Disorder (BPD) Diagnosis** (n = 32)	**BPD – High Lethality** (n = 36)	**BPD – Low Lethality** (n = 49)	**Omnibus *p* Value (Significant post-hoc group differences)**
Male sex, No (%)	14 (25.9)	8 (25.0)	4 (11.1)	10 (20.4)	0.36
Age, y (sd)	30.5 (9.0)	29.3 (6.5)	33.1 (11.4)	29.6 (8.8)	0.26
Race (American Indian or Alaska Native/Asian/Black/Caucasian/Multi-race/Native Hawaiian or Pacific Islander/Prefer not to answer)	0/8/9/33/4/0/0	0/0/3/24/4/1/0	0/0/4/30/1/0/1	0/2/2/38/3/0/4	0.05 (Caucasian vs. all others)
Educational level, y (sd)	16.4 (2.7)	15.1 (2.0)	14.8 (1.7)	15.1 (1.8)	0.004 (HC > BPD – High Lethality, BPD – Low Lethality)
Premorbid IQ estimate, WTAR[1] (sd)	111.0 (9.8)	113.0 (10.5)	110.0 (10.3)	109.0 (10.6)	0.40
Executive interview[15] score (sd)	4.06 (3.04)	5.32 (2.83)	4.08 (2.82)	4.71 (2.03)	0.16
Hamilton Rating Scale for Depression[16] (17 items) score	2.88 (2.40)	18.0 (6.57)	18.0 (6.11)	17.6 (5.95)	<.001 (HC < all others)
Antidepressant exposure (past 6 months; The Antidepressant Treatment History Form[17])	NA	19	25	29	0.58
Lifetime substance use, No.	NA	17	12	21	0.26
Lifetime anxiety, No.	NA	28	24	41	0.07
Lifetime depression, No.	NA	27	27	43	0.29

Note: The last column provides *p* values from omnibus likelihood ratio chi-squared tests comparing mean differences across groups. In the brackets, we report Bonferroni-corrected post-hoc pairwise group comparisons.

© 2024 Tsypes A et al. *JAMA Psychiatry.*

eTable 2. Demographic and clinical characterization of the MDD sample

Characteristic	**Nonpsychiatric Controls** (n = 43)	**Nonsuicidal Depressed** (n = 32)	**Depressed with Suicidal Ideation** (n = 31)	**Depressed Suicide Attempters – High Lethality** (n = 13)	**Depressed Suicide Attempters – Low Lethality** (n = 24)	**Omnibus *p* Value (Bonferroni post-hocs for significant effects)**
Male sex, No (%)	20 (46.5)	17 (53.1)	13 (41.9)	5 (38.5)	7 (29.2)	0.47
Age, y (sd)	63.3 (8.2)	61.9 (6.7)	61.5 (5.0)	59.8 (5.2)	61.8 (7.1)	0.53
Race (Asian/Black/Caucasian/Multi-race)	0/4/39/0	0/5/26/1	1/2/28/0	0/0/12/1	1/4/18/1	0.47 (Caucasian vs. all others)
Educational level, y (sd)	16.5 (2.8)	15.7 (2.3)	15.4 (1.9)	14.5 (3.0)	14.5 (2.7)	0.01 (HC > Attempters - Low Lethality)
Premorbid IQ estimate, WTAR[1] (sd)	111.7 (9.7)	108.5 (13.7)	113.8 (11.2)	105.8 (10.1)	103.5 (12.0)	0.01 (Ideators > Attempters - Low Lethality)
Dementia rating scale score	138.1 (3.5)	135.6 (5.0)	136.9 (3.7)	134.9 (4.8)	135.6 (3.8)	0.03 (post-hocs n.s.)
Executive interview[15] score (sd)	4.95 (2.45)	6.14 (3.42)	5.79 (2.73)	6.00 (2.58)	6.65 (3.49)	0.21
Physical illness burden (The cumulative Illness Rating Scale-Geriatric[18])	4.6 (3.1)	8.7 (4.6)	9.5 (4.8)	8.2 (4.0)	9.3 (4.9)	<.001 (HC < Nonsuicidal Depressed, Ideators, Attempters – Low Lethality)
Hamilton Rating Scale for Depression[16] (17 items) score	2.0 (2.4)	13.0 (4.4)	15.8 (6.7)	14.2 (5.7)	14.9 (8.6)	<.001 (HC < all other groups)
Beck Hopelessness Scale[19] score	1.2 (1.3)	5.9 (5.1)	11.6 (6.2)	7.8 (6.2)	9.0 (6.7)	<.001 (HC < all other groups, Nonsuicidal

© 2024 Tsypes A et al. *JAMA Psychiatry.*

						Depressed < Ideators)
Antidepressant exposure (past 6 months; The Antidepressant Treatment History Form[17])	NA	21	24	11	20	0.25
Lifetime substance use, No.	NA	14	16	8	17	0.22
Lifetime anxiety, No.	NA	16	19	12	18	0.03 (post-hocs n.s.)

Note: The last column provides *p* values from omnibus likelihood ratio chi-squared tests comparing mean differences across groups. In the brackets, we report Bonferroni-corrected post-hoc pairwise group comparisons.

© 2024 Tsypes A et al. *JAMA Psychiatry.*

eTable 3. Model fits by diagnostic groups

Model	Nonpsychiatric Controls (n = 54)	BPD (n = 32)	BPD – High Lethality (n = 36)	BPD – Low Lethality (n = 49)	Full sample (n = 171)
Uncertainty-sensitive Reinforcement Learning, N, (% best fit)	7 (0.13)	6 (0.19)	5 (0.14)	11 (0.22)	29 (0.17)
Traditional Reinforcement Learning, N, (% best fit)	1 (0.02)	0 (0)	0 (0)	0 (0)	1 (0.01)
Information-compressing Reinforcement Learning, N, (% best fit)	46 (0.85)	26 (0.81)	31 (0.86)	38 (0.78)	141 (0.82)

© 2024 Tsypes A et al. *JAMA Psychiatry.*

eTable 4. BPD sample: Exploration and exploitation on the Clock task

term	coefficient	SE	t-value	95% CI low	95% CI high
RT lag	0.29***	0.01	24.22	0.27	0.31
reward lag	0.17***	0.02	8.46	0.13	0.21
BPD-LL	0.02	0.04	0.48	-0.06	0.10
BPD-NON	0.02	0.05	0.44	-0.07	0.11
HC	0.01	0.04	0.28	-0.07	0.09
RT vmax	0.18***	0.01	14.81	0.15	0.20
trial	0.03	0.02	1.90	-0.00	0.06
RT lag:reward lag	0.43***	0.02	23.15	0.40	0.47
RT lag:BPD-LL	-0.07***	0.02	-4.32	-0.10	-0.04
RT lag:BPD-NON	-0.01	0.02	-0.72	-0.05	0.02
RT lag:HC	0.03	0.02	1.88	-0.00	0.06
reward lag:BPD-LL	0.00	0.03	0.15	-0.05	0.06
reward lag:BPD-NON	-0.02	0.03	-0.67	-0.08	0.04
reward lag:HC	0.08**	0.03	3.07	0.03	0.13
RT vmax:trial	0.05**	0.02	2.58	0.01	0.09
BPD-LL:RT vmax	-0.02	0.02	-1.16	-0.05	0.01
BPD-NON:RT vmax	-0.01	0.02	-0.42	-0.04	0.03
HC:RT vmax	-0.03*	0.02	-2.31	-0.06	-0.01
BPD-LL:trial	0.00	0.02	0.05	-0.04	0.04
BPD-NON:trial	0.01	0.02	0.44	-0.04	0.06
HC:trial	-0.04*	0.02	-2.12	-0.09	-0.00
RT lag:trial	-0.04*	0.02	-2.18	-0.08	-0.00
**RT lag:reward lag:BPD-LL**	**0.07****	**0.02**	**2.81**	**0.02**	**0.12**
**RT lag:reward lag:BPD-NON**	**0.07****	**0.03**	**2.64**	**0.02**	**0.13**
**RT lag:reward lag:HC**	**0.11*****	**0.02**	**4.46**	**0.06**	**0.16**
BPD-LL:RT vmax:trial	***-0.04***	***0.03***	***-1.49***	***-0.09***	***0.01***
BPD-NON:RT vmax:trial	***0.00***	***0.03***	***0.18***	***-0.05***	***0.06***
HC:RT vmax:trial	***0.00***	***0.02***	***0.14***	***-0.04***	***0.05***

© 2024 Tsypes A et al. *JAMA Psychiatry.*

term	coefficient	SE	t-value	95% CI low	95% CI high
RT lag:BPD-LL:trial	0.03	0.03	1.01	-0.02	0.08
RT lag:BPD-NON:trial	0.03	0.03	0.94	-0.03	0.08
RT lag:HC:trial	0.01	0.03	0.30	-0.04	0.06

Note: *p<0.05; ** p<0.01; ***p<0.001. Estimates are from a multilevel linear regression model fitted to trial-level data using restricted maximum likelihood, accounting for random intercepts for each subject and run. BPD-HL (high-lethality attempters) and reward omission were the references. Dependent variable: Response time (RT) on the current trial. SE: standard error; CI: confidence interval.

RT lag: RT lagged by one trial; reward lag: Reward vs. omission feedback outcome lagged by one trial; BPD: Borderline personality disorder; BPD-LL: Low-lethality suicide attempters diagnosed with BPD (vs. high-lethality attempters diagnosed with BPD); BPD-NON: Non-attempters diagnosed with BPD (vs. high-lethality attempters diagnosed with BPD); HC: Healthy controls (vs. high-lethality attempters diagnosed with BPD); RT vmax: The most rewarded RT learned on the task to date; trial: Trial within current condition. **The bolded terms represent between-group differences in exploration (RT lag:reward lag:GROUP) and exploitation** (***GROUP:RT vmax:trial***).

© 2024 Tsypes A et al. *JAMA Psychiatry.*

eTable 5. BPD sample sensitivity analysis: Levels of depressive symptoms (excluding healthy controls)

term	coefficient	SE	t-value	95% CI low	95% CI high
RT lag	0.29***	0.01	24.50	0.27	0.31
reward lag	0.18***	0.02	9.01	0.14	0.22
BPD-LL	0.02	0.04	0.49	-0.06	0.10
BPD-NON	0.02	0.05	0.48	-0.07	0.12
HRSD17	-0.01	0.02	-0.59	-0.04	0.02
RT vmax	0.18***	0.01	14.91	0.15	0.20
trial	0.03*	0.02	1.99	0.00	0.06
RT lag:reward lag	0.43***	0.02	23.13	0.39	0.46
RT lag:BPD-LL	-0.07***	0.02	-4.41	-0.10	-0.04
RT lag:BPD-NON	-0.01	0.02	-0.68	-0.04	0.02
reward lag:BPD-LL	-0.00	0.03	-0.18	-0.06	0.05
reward lag:BPD-NON	-0.03	0.03	-1.18	-0.09	0.02
RT lag:HRSD17	-0.01	0.01	-0.85	-0.02	0.01
reward lag:HRSD17	0.02	0.01	1.81	-0.00	0.04
RT vmax:trial	0.05**	0.02	2.64	0.01	0.09
BPD-LL:RT vmax	-0.02	0.02	-1.18	-0.05	0.01
BPD-NON:RT vmax	-0.01	0.02	-0.43	-0.04	0.03
BPD-LL:trial	0.00	0.02	0.00	-0.04	0.04
BPD-NON:trial	0.01	0.02	0.33	-0.04	0.05
RT lag:trial	-0.05*	0.02	-2.40	-0.09	-0.01
RT lag:reward lag:BPD-LL	0.08**	0.02	3.11	0.03	0.13
RT lag:reward lag:BPD-NON	0.07**	0.03	2.73	0.02	0.13
**RT lag:reward lag:HRSD17**	**-0.02***	**0.01**	**-2.16**	**-0.04**	**-0.00**
BPD-LL:RT vmax:trial	-0.04	0.02	-1.52	-0.09	0.01
BPD-NON:RT vmax:trial	0.00	0.03	0.06	-0.05	0.06
RT lag:BPD-LL:trial	0.03	0.03	1.17	-0.02	0.08
RT lag:BPD-NON:trial	0.03	0.03	1.16	-0.02	0.09

© 2024 Tsypes A et al. *JAMA Psychiatry.*

Note: $^{*}p<0.05$; $^{**}p<0.01$; $^{***}p<0.001$.

Estimates are from a multilevel linear regression model fitted to trial-level data using restricted maximum likelihood, accounting for random intercepts for each subject and run. BPD-HL (high-lethality attempters) and reward omission were the references. Dependent variable: Response time (RT) on the current trial. SE: standard error; CI: confidence interval.

RT lag: RT lagged by one trial; reward lag: Reward vs. omission feedback outcome lagged by one trial; BPD: Borderline personality disorder; BPD-LL: Low-lethality suicide attempters diagnosed with BPD (vs. high-lethality attempters diagnosed with BPD); BPD-NON: Non-attempters diagnosed with BPD (vs. high-lethality attempters diagnosed with BPD); HC: Healthy controls (vs. high-lethality attempters diagnosed with BPD); RT vmax: The most rewarded RT learned on the task to date; trial: Trial within current condition; **HRSD17: Hamilton Rating Scale for Depression (17 items). The bolded term represents the critical (for this sensitivity analysis) RT lag:reward lag:HRSD17 interaction, accounting for the effects of depressive symptoms on exploration.**

© 2024 Tsypes A et al. *JAMA Psychiatry.*

eTable 6. BPD sample sensitivity analysis: Suicide attempt recency

term	coefficient	SE	t-value	95% CI low	95% CI high
RT lag	0.30***	0.01	24.59	0.28	0.33
reward lag	0.17***	0.02	8.33	0.13	0.22
BPD-LL	0.04	0.05	0.79	-0.06	0.13
SA recency	-0.00	0.02	-0.06	-0.05	0.04
RT vmax	0.18***	0.01	14.35	0.15	0.20
trial	0.03	0.02	1.86	-0.00	0.06
RT lag:reward lag	0.44***	0.02	22.86	0.40	0.48
RT lag:BPD-LL	-0.07***	0.02	-4.52	-0.10	-0.04
reward lag:BPD-LL	-0.00	0.03	-0.14	-0.06	0.05
RT lag:SA recency	-0.02*	0.01	-2.48	-0.03	-0.00
reward lag:SA recency	0.02	0.01	1.59	-0.01	0.05
RT vmax:trial	0.06**	0.02	2.75	0.02	0.09
BPD-LL:RT vmax	-0.02	0.02	-1.18	-0.05	0.01
BPD-LL:trial	0.00	0.02	0.16	-0.04	0.05
RT lag:trial	-0.04*	0.02	-2.09	-0.08	-0.00
RT lag:reward lag:BPD-LL	0.05*	0.03	2.03	0.00	0.10
**RT lag:reward lag:SA recency**	**0.07*** **	**0.01**	**5.32**	**0.05**	**0.10**
BPD-LL:RT vmax:trial	-0.05	0.03	-1.72	-0.10	0.01
RT lag:BPD-LL:trial	0.02	0.03	0.81	-0.03	0.07

Note: *p<0.05; ** p<0.01; ***p<0.001. Estimates are from a multilevel linear regression model fitted to trial-level data using restricted maximum likelihood, accounting for random intercepts for each subject and run. BPD-HL (high-lethality attempters) and reward omission were the references. Dependent variable: Response time (RT) on the current trial. SE: standard error; CI: confidence interval.

RT lag: RT lagged by one trial; reward lag: Reward vs. omission feedback outcome lagged by one trial; BPD: Borderline personality disorder; BPD-LL: Low-lethality suicide attempters diagnosed with BPD (vs. high-lethality attempters diagnosed with BPD); BPD-NON: Non-attempters diagnosed with BPD (vs. high-lethality attempters diagnosed with BPD); HC: Healthy controls (vs. high-lethality attempters diagnosed with BPD); RT vmax: The most rewarded RT learned on the task to date; trial: Trial within current condition; **SA recency: Time between the most recent suicide attempt and Clock task completion. The bolded term represents the critical (for this sensitivity analysis) RT lag:reward lag:SA recency interaction, accounting for the effects of suicide attempt recency on exploration.**

© 2024 Tsypes A et al. *JAMA Psychiatry.*

eTable 7. BPD sample sensitivity analysis: Impulsivity

term	coefficient	SE	t-value	95% CI low	95% CI high
RT lag	0.30***	0.01	22.88	0.27	0.32
reward lag	0.20***	0.02	9.11	0.16	0.24
BPD-LL	0.03	0.04	0.79	-0.05	0.12
BPD-NON	0.03	0.05	0.64	-0.06	0.12
HC	0.08	0.05	1.49	-0.03	0.19
pid5_disinhibition	0.05*	0.02	2.09	0.00	0.09
RT vmax	0.18***	0.01	15.02	0.16	0.21
trial	0.03	0.02	1.92	-0.00	0.07
RT lag:reward lag	0.47***	0.02	22.54	0.43	0.51
RT lag:BPD-LL	-0.07***	0.02	-4.34	-0.10	-0.04
RT lag:BPD-NON	-0.02	0.02	-1.08	-0.05	0.02
RT lag:HC	0.02	0.02	0.95	-0.02	0.06
reward lag:BPD-LL	-0.01	0.03	-0.22	-0.06	0.05
reward lag:BPD-NON	-0.03	0.03	-1.04	-0.09	0.03
reward lag:HC	0.02	0.04	0.59	-0.05	0.09
RT lag: disinhibition	-0.01	0.01	-0.77	-0.02	0.01
reward lag: disinhibition	-0.03*	0.01	-2.46	-0.06	-0.01
RT vmax:trial	0.06**	0.02	2.91	0.02	0.10
BPD-LL:RT vmax	-0.03	0.02	-1.62	-0.06	0.01
BPD-NON:RT vmax	-0.01	0.02	-0.84	-0.05	0.02
HC:RT vmax	-0.04**	0.02	-2.62	-0.07	-0.01
BPD-LL:trial	0.00	0.02	0.08	-0.04	0.04
BPD-NON:trial	0.01	0.02	0.37	-0.04	0.06
HC:trial	-0.04	0.02	-1.89	-0.08	0.00
RT lag:trial	-0.04*	0.02	-2.10	-0.09	-0.00
RT lag:reward lag:BPD-LL	0.07**	0.03	2.78	0.02	0.12
RT lag:reward lag:BPD-NON	0.07*	0.03	2.44	0.01	0.12
RT lag:reward lag:HC	0.02	0.03	0.68	-0.04	0.09
**RT lag:reward lag: disinhibition**	**-0.05*** **	**0.01**	**-3.67**	**-0.07**	**-0.02**

© 2024 Tsypes A et al. *JAMA Psychiatry.*

term	coefficient	SE	t-value	95% CI low	95% CI high
BPD-LL:RT vmax:trial	-0.04	0.03	-1.71	-0.09	0.01
BPD-NON:RT vmax:trial	-0.00	0.03	-0.07	-0.06	0.05
HC:RT vmax:trial	-0.00	0.03	-0.05	-0.05	0.05
RT lag:BPD-LL:trial	0.03	0.03	0.94	-0.03	0.08
RT lag:BPD-NON:trial	0.03	0.03	0.93	-0.03	0.08
RT lag:HC:trial	0.00	0.03	0.15	-0.05	0.06

Note: *p<0.05; ** p<0.01; ***p<0.001. Estimates are from a multilevel linear regression model fitted to trial-level data using restricted maximum likelihood, accounting for random intercepts for each subject and run. BPD-HL (high-lethality attempters) and reward omission were the references. Dependent variable: Response time (RT) on the current trial. SE: standard error; CI: confidence interval.

RT lag: RT lagged by one trial; reward lag: Reward vs. omission feedback outcome lagged by one trial; BPD: Borderline personality disorder; BPD-LL: Low-lethality suicide attempters diagnosed with BPD (vs. high-lethality attempters diagnosed with BPD); BPD-NON: Non-attempters diagnosed with BPD (vs. high-lethality attempters diagnosed with BPD); HC: Healthy controls (vs. high-lethality attempters diagnosed with BPD); RT vmax: The most rewarded RT learned on the task to date; trial: Trial within current condition; **disinhibition: PID5 disinhibition domain score. The bolded term represents the critical (for this sensitivity analysis) RT lag:reward lag: disinhibition interaction, accounting for the effects of disinhibition domain on exploration.**

© 2024 Tsypes A et al. *JAMA Psychiatry.*

eTable 8. BPD sample sensitivity analysis: Medication exposure to antidepressants (excluding healthy controls)

term	coefficient	SE	t-value	95% CI low	95% CI high
RT lag	0.27***	0.01	19.46	0.25	0.30
reward lag	0.19***	0.02	7.77	0.14	0.24
BPD-LL	0.02	0.04	0.42	-0.07	0.10
BPD-NON	0.02	0.05	0.37	-0.08	0.11
antidepressants	-0.01	0.01	-0.50	-0.03	0.02
RT vmax	0.18***	0.01	14.93	0.15	0.20
trial	0.03	0.02	1.93	-0.00	0.06
RT lag:reward lag	0.46***	0.02	20.35	0.42	0.51
RT lag:BPD-LL	-0.06***	0.02	-4.11	-0.09	-0.03
RT lag:BPD-NON	-0.01	0.02	-0.48	-0.04	0.03
reward lag:BPD-LL	-0.00	0.03	-0.02	-0.05	0.05
reward lag:BPD-NON	-0.02	0.03	-0.80	-0.08	0.03
RT lag:antidepressants	0.01*	0.00	1.98	0.00	0.01
reward lag:antidepressants	-0.01	0.01	-1.68	-0.02	0.00
RT vmax:trial	0.05*	0.02	2.57	0.01	0.09
BPD-LL:RT vmax	-0.02	0.02	-1.24	-0.05	0.01
BPD-NON:RT vmax	-0.01	0.02	-0.51	-0.04	0.02
BPD-LL:trial	0.00	0.02	0.01	-0.04	0.04
BPD-NON:trial	0.01	0.02	0.41	-0.04	0.06
RT lag:trial	-0.04*	0.02	-2.13	-0.08	-0.00
RT lag:reward lag:BPD-LL	0.07**	0.03	2.71	0.02	0.12
RT lag:reward lag:BPD-NON	0.07*	0.03	2.44	0.01	0.12
**RT lag:reward lag:antidepressants**	**-0.01***	**0.01**	**-2.23**	**-0.03**	**-0.00**
BPD-LL:RT vmax:trial	-0.04	0.03	-1.45	-0.09	0.01
BPD-NON:RT vmax:trial	0.01	0.03	0.19	-0.05	0.06
RT lag:BPD-LL:trial	0.02	0.03	0.95	-0.03	0.08
RT lag:BPD-NON:trial	0.03	0.03	0.93	-0.03	0.08

© 2024 Tsypes A et al. *JAMA Psychiatry.*

Note: *p<0.05; ** p<0.01; ***p<0.001. Estimates are from a multilevel linear regression model fitted to trial-level data using restricted maximum likelihood, accounting for random intercepts for each subject and run. BPD-HL (high-lethality attempters) and reward omission were the references. Dependent variable: Response time (RT) on the current trial. SE: standard error; CI: confidence interval.

RT lag: RT lagged by one trial; reward lag: Reward vs. omission feedback outcome lagged by one trial; BPD: Borderline personality disorder; BPD-LL: Low-lethality suicide attempters diagnosed with BPD (vs. high-lethality attempters diagnosed with BPD); BPD-NON: Non-attempters diagnosed with BPD (vs. high-lethality attempters diagnosed with BPD); HC: Healthy controls (vs. high-lethality attempters diagnosed with BPD); RT vmax: The most rewarded RT learned on the task to date; trial: Trial within current condition; **antidepressants: Exposure to antidepressants in the past 6 months. The bolded term represents the critical (for this sensitivity analysis) RT lag:reward lag:antidepressants interaction, accounting for the effects of antidepressant use on exploration.**

© 2024 Tsypes A et al. *JAMA Psychiatry.*

eTable 9. BPD sample sensitivity analysis: Medication exposure to opioids (excluding healthy controls)

term	coefficient	SE	t-value	95% CI low	95% CI high
RT lag	0.29***	0.01	23.68	0.26	0.31
reward lag	0.17***	0.02	8.36	0.13	0.21
BPD-LL	0.01	0.04	0.33	-0.07	0.10
BPD-NON	0.01	0.05	0.30	-0.08	0.11
opioid	-0.21	0.20	-1.07	-0.60	0.18
RT vmax	0.18***	0.01	14.81	0.15	0.20
trial	0.03	0.02	1.87	-0.00	0.06
RT lag:reward lag	0.44***	0.02	22.83	0.40	0.47
RT lag:BPD-LL	-0.06***	0.02	-4.12	-0.09	-0.03
RT lag:BPD-NON	-0.01	0.02	-0.58	-0.04	0.02
reward lag:BPD-LL	0.00	0.03	0.14	-0.05	0.06
reward lag:BPD-NON	-0.02	0.03	-0.68	-0.08	0.04
RT lag:opioids	0.06	0.07	0.89	-0.07	0.19
reward lag:opioids	-0.03	0.15	-0.20	-0.33	0.27
RT vmax:trial	0.05**	0.02	2.62	0.01	0.09
BPD-LL:RT vmax	-0.02	0.02	-1.16	-0.05	0.01
BPD-NON:RT vmax	-0.01	0.02	-0.43	-0.04	0.03
BPD-LL:trial	0.00	0.02	0.07	-0.04	0.04
BPD-NON:trial	0.01	0.02	0.45	-0.04	0.06
RT lag:trial	-0.04*	0.02	-2.22	-0.08	-0.01
RT lag:reward lag:BPD-LL	0.07**	0.03	2.72	0.02	0.12
RT lag:reward lag:BPD-NON	0.07*	0.03	2.56	0.02	0.13
**RT lag:reward lag:opioids**	**-0.06**	**0.13**	**-0.44**	**-0.31**	**0.19**
BPD-LL:RT vmax:trial	-0.04	0.03	-1.50	-0.09	0.01
BPD-NON:RT vmax:trial	0.00	0.03	0.16	-0.05	0.06
RT lag:BPD-LL:trial	0.03	0.03	1.04	-0.02	0.08
RT lag:BPD-NON:trial	0.03	0.03	0.97	-0.03	0.08

© 2024 Tsypes A et al. *JAMA Psychiatry.*

Note: *p<0.05; ** p<0.01; ***p<0.001. Estimates are from a multilevel linear regression model fitted to trial-level data using restricted maximum likelihood, accounting for random intercepts for each subject and run. BPD-HL (high-lethality attempters) and reward omission were the references. Dependent variable: Response time (RT) on the current trial. SE: standard error; CI: confidence interval.

RT lag: RT lagged by one trial; reward lag: Reward vs. omission feedback outcome lagged by one trial; BPD: Borderline personality disorder; BPD-LL: Low-lethality suicide attempters diagnosed with BPD (vs. high-lethality attempters diagnosed with BPD); BPD-NON: Non-attempters diagnosed with BPD (vs. high-lethality attempters diagnosed with BPD); HC: Healthy controls (vs. high-lethality attempters diagnosed with BPD); RT vmax: The most rewarded RT learned on the task to date; trial: Trial within current condition; **opioids: Exposure to opioids in the past 6 months. The bolded term represents the critical (for this sensitivity analysis) RT lag:reward lag:opioids interaction, accounting for the effects of opioid use on exploration.**

© 2024 Tsypes A et al. *JAMA Psychiatry.*

eTable 10. BPD sample sensitivity analysis: Medication exposure to sedatives or hypnotics (excluding healthy controls)

term	coefficient	SE	t-value	95% CI low	95% CI high
RT lag	0.29***	0.01	24.20	0.27	0.31
reward lag	0.17***	0.02	8.45	0.13	0.21
BPD-LL	0.02	0.04	0.49	-0.06	0.10
BPD-NON	0.02	0.05	0.39	-0.07	0.11
sedhyp	0.08	0.20	0.42	-0.31	0.47
RT vmax	0.18***	0.01	14.84	0.15	0.20
trial	0.03	0.02	1.89	-0.00	0.06
RT lag:reward lag	0.43***	0.02	23.09	0.40	0.47
RT lag:BPD-LL	-0.07***	0.02	-4.31	-0.10	-0.04
RT lag:BPD-NON	-0.01	0.02	-0.67	-0.05	0.02
reward lag:BPD-LL	0.00	0.03	0.15	-0.05	0.06
reward lag:BPD-NON	-0.02	0.03	-0.66	-0.08	0.04
RT lag:sedhyp	-0.02	0.06	-0.31	-0.14	0.10
reward lag:sedhyp	0.01	0.13	0.06	-0.25	0.27
RT vmax:trial	0.05**	0.02	2.60	0.01	0.09
BPD-LL:RT vmax	-0.02	0.02	-1.16	-0.05	0.01
BPD-NON:RT vmax	-0.01	0.02	-0.44	-0.04	0.03
BPD-LL:trial	0.00	0.02	0.05	-0.04	0.04
BPD-NON:trial	0.01	0.02	0.42	-0.04	0.06
RT lag:trial	-0.04*	0.02	-2.18	-0.08	-0.00
RT lag:reward lag:BPD-LL	0.07**	0.03	2.80	0.02	0.12
RT lag:reward lag:BPD-NON	0.08**	0.03	2.68	0.02	0.13
**RT lag:reward lag:sedhyp**	**-0.07**	**0.12**	**-0.57**	**-0.29**	**0.16**
BPD-LL:RT vmax:trial	-0.04	0.03	-1.48	-0.09	0.01
BPD-NON:RT vmax:trial	0.01	0.03	0.18	-0.05	0.06
RT lag:BPD-LL:trial	0.03	0.03	1.01	-0.02	0.08
RT lag:BPD-NON:trial	0.03	0.03	0.94	-0.03	0.08

© 2024 Tsypes A et al. *JAMA Psychiatry.*

Note: *p<0.05; ** p<0.01; ***p<0.001. Estimates are from a multilevel linear regression model fitted to trial-level data using restricted maximum likelihood, accounting for random intercepts for each subject and run. BPD-HL (high-lethality attempters) and reward omission were the references. Dependent variable: Response time (RT) on the current trial. SE: standard error; CI: confidence interval.

RT lag: RT lagged by one trial; reward lag: Reward vs. omission feedback outcome lagged by one trial; BPD: Borderline personality disorder; BPD-LL: Low-lethality suicide attempters diagnosed with BPD (vs. high-lethality attempters diagnosed with BPD); BPD-NON: Non-attempters diagnosed with BPD (vs. high-lethality attempters diagnosed with BPD); HC: Healthy controls (vs. high-lethality attempters diagnosed with BPD); RT vmax: The most rewarded RT learned on the task to date; trial: Trial within current condition; **sedhyps: Exposure to sedatives or hypnotics in the past 6 months. The bolded term represents the critical (for this sensitivity analysis) RT lag:reward lag:sedhyp interaction, accounting for the effects of sedatives/hypnotics use on exploration.**

© 2024 Tsypes A et al. *JAMA Psychiatry.*

eTable 11. BPD sample sensitivity analysis: Medication exposure to antipsychotics (excluding healthy controls)

term	coefficient	SE	t-value	95% CI low	95% CI high
RT lag	0.27***	0.01	21.64	0.25	0.30
reward lag	0.18***	0.02	8.21	0.13	0.22
BPD-LL	0.02	0.04	0.57	-0.06	0.11
BPD-NON	0.02	0.05	0.51	-0.07	0.12
antipsychotic	0.03	0.05	0.60	-0.07	0.14
RT vmax	0.18***	0.01	15.01	0.15	0.20
trial	0.03	0.02	1.86	-0.00	0.06
RT lag:reward lag	0.45***	0.02	22.46	0.41	0.49
RT lag:BPD-LL	-0.06***	0.02	-3.64	-0.09	-0.03
RT lag:BPD-NON	-0.00	0.02	-0.19	-0.04	0.03
reward lag:BPD-LL	0.00	0.03	0.02	-0.05	0.05
reward lag:BPD-NON	-0.02	0.03	-0.75	-0.08	0.04
RT lag:antipsychotics	0.06***	0.02	3.30	0.02	0.09
reward lag:antipsychotics	-0.03	0.03	-0.93	-0.10	0.04
RT vmax:trial	0.05**	0.02	2.64	0.01	0.09
BPD-LL:RT vmax	-0.02	0.02	-1.31	-0.05	0.01
BPD-NON:RT vmax	-0.01	0.02	-0.64	-0.04	0.02
BPD-LL:trial	0.00	0.02	0.05	-0.04	0.04
BPD-NON:trial	0.01	0.02	0.48	-0.04	0.06
RT lag:trial	-0.05*	0.02	-2.26	-0.09	-0.01
RT lag:reward lag:BPD-LL	0.06*	0.03	2.48	0.01	0.11
RT lag:reward lag:BPD-NON	0.06*	0.03	2.27	0.01	0.12
**RT lag:reward lag:antipsychotics**	**-0.06***	**0.03**	**-2.11**	**-0.12**	**-0.00**
BPD-LL:RT vmax:trial	-0.04	0.03	-1.48	-0.09	0.01
BPD-NON:RT vmax:trial	0.00	0.03	0.12	-0.05	0.06
RT lag:BPD-LL:trial	0.03	0.03	1.03	-0.02	0.08
RT lag:BPD-NON:trial	0.03	0.03	1.06	-0.03	0.08

Note: $^{*}p<0.05$; $^{**}p<0.01$; $^{***}p<0.001$. Estimates are from a multilevel linear regression model fitted to trial-level data using restricted maximum likelihood, accounting for random intercepts for each subject and run. BPD-HL (high-lethality attempters)

© 2024 Tsypes A et al. *JAMA Psychiatry.*

and reward omission were the references. Dependent variable: Response time (RT) on the current trial. SE: standard error; CI: confidence interval.

RT lag: RT lagged by one trial; reward lag: Reward vs. omission feedback outcome lagged by one trial; BPD: Borderline personality disorder; BPD-LL: Low-lethality suicide attempters diagnosed with BPD (vs. high-lethality attempters diagnosed with BPD); BPD-NON: Non-attempters diagnosed with BPD (vs. high-lethality attempters diagnosed with BPD); HC: Healthy controls (vs. high-lethality attempters diagnosed with BPD); RT vmax: The most rewarded RT learned on the task to date; trial: Trial within current condition; **antipsychotics: Exposure to sedatives or antipsychotics in the past 6 months. The bolded term represents the critical (for this sensitivity analysis) RT lag:reward lag: antipsychotics interaction, accounting for the effects of antipsychotics use on exploration.**

© 2024 Tsypes A et al. *JAMA Psychiatry.*

eTable 12. BPD sample sensitivity analysis: Estimated premorbid IQ

term	coefficient	SE	t-value	95% CI low	95% CI high
RT lag	0.30***	0.01	23.84	0.28	0.33
reward lag	0.16***	0.02	7.69	0.12	0.20
BPD-LL	0.04	0.04	0.89	-0.05	0.12
BPD-NON	0.02	0.05	0.35	-0.08	0.11
HC	0.02	0.04	0.35	-0.07	0.10
WTAR	0.03*	0.02	2.11	0.00	0.06
RT vmax	0.17***	0.01	13.73	0.15	0.20
trial	0.04*	0.02	2.07	0.00	0.07
RT lag:reward lag	0.43***	0.02	21.61	0.39	0.47
RT lag:BPD-LL	-0.08***	0.02	-4.93	-0.11	-0.05
RT lag:BPD-NON	-0.03	0.02	-1.43	-0.06	0.01
RT lag:HC	0.01	0.02	0.86	-0.02	0.05
reward lag:BPD-LL	0.00	0.03	0.13	-0.05	0.06
reward lag:BPD-NON	-0.01	0.03	-0.21	-0.07	0.05
reward lag:HC	0.09***	0.03	3.36	0.04	0.15
RT lag:WTAR	0.00	0.01	0.12	-0.01	0.01
reward lag:WTAR	-0.03**	0.01	-2.68	-0.05	-0.01
RT vmax:trial	0.06**	0.02	2.97	0.02	0.10
BPD-LL:RT vmax	-0.02	0.02	-1.09	-0.05	0.01
BPD-NON:RT vmax	-0.00	0.02	-0.27	-0.04	0.03
HC:RT vmax	-0.03	0.02	-1.84	-0.06	0.00
BPD-LL:trial	-0.01	0.02	-0.33	-0.05	0.04
BPD-NON:trial	0.01	0.02	0.22	-0.04	0.05
HC:trial	-0.04	0.02	-1.82	-0.09	0.00
RT lag:trial	-0.05*	0.02	-2.31	-0.09	-0.01
RT lag:reward lag:BPD-LL	0.08**	0.03	3.08	0.03	0.13
RT lag:reward lag:BPD-NON	0.08**	0.03	2.75	0.02	0.13
RT lag:reward lag:HC	0.12***	0.03	4.57	0.07	0.17

© 2024 Tsypes A et al. *JAMA Psychiatry.*

term	coefficient	SE	t-value	95% CI low	95% CI high
**RT lag:reward lag:WTAR**	**0.01**	**0.01**	**1.48**	**-0.00**	**0.03**
BPD-LL:RT vmax:trial	-0.05	0.03	-1.91	-0.10	0.00
BPD-NON:RT vmax:trial	-0.01	0.03	-0.21	-0.06	0.05
HC:RT vmax:trial	-0.01	0.03	-0.29	-0.06	0.04
RT lag:BPD-LL:trial	0.03	0.03	1.23	-0.02	0.09
RT lag:BPD-NON:trial	0.03	0.03	1.10	-0.03	0.09
RT lag:HC:trial	0.02	0.03	0.59	-0.04	0.07

Note: *p<0.05; ** p<0.01; ***p<0.001. Estimates are from a multilevel linear regression model fitted to trial-level data using restricted maximum likelihood, accounting for random intercepts for each subject and run. BPD-HL (high-lethality attempters) and reward omission were the references. Dependent variable: Response time (RT) on the current trial. SE: standard error; CI: confidence interval.

RT lag: RT lagged by one trial; reward lag: Reward vs. omission feedback outcome lagged by one trial; BPD: Borderline personality disorder; BPD-LL: Low-lethality suicide attempters diagnosed with BPD (vs. high-lethality attempters diagnosed with BPD); BPD-NON: Non-attempters diagnosed with BPD (vs. high-lethality attempters diagnosed with BPD); HC: Healthy controls (vs. high-lethality attempters diagnosed with BPD); RT vmax: The most rewarded RT learned on the task to date; trial: Trial within current condition; **WTAR: Estimated premorbid IQ. The bolded term represents the critical (for this sensitivity analysis) RT lag:reward lag: WTAR interaction, accounting for the effects of estimated premorbid IQ on exploration.**

© 2024 Tsypes A et al. *JAMA Psychiatry.*

eTable 13. BPD sample sensitivity analysis: Executive function

term	coefficient	SE	t-value	95% CI low	95% CI high
RT lag	0.30***	0.01	24.21	0.28	0.33
reward lag	0.17***	0.02	8.13	0.13	0.21
BPD-LL	0.05	0.04	1.03	-0.04	0.13
BPD-NON	0.02	0.05	0.40	-0.08	0.11
HC	0.01	0.04	0.28	-0.07	0.10
EXIT	-0.01	0.02	-0.47	-0.04	0.02
RT vmax	0.17***	0.01	13.77	0.15	0.20
trial	0.03	0.02	1.92	-0.00	0.07
RT lag:reward lag	0.42***	0.02	21.79	0.39	0.46
RT lag:BPD-LL	-0.08***	0.02	-4.87	-0.11	-0.05
RT lag:BPD-NON	-0.03	0.02	-1.53	-0.06	0.01
RT lag:HC	0.01	0.02	0.93	-0.02	0.05
reward lag:BPD-LL	0.00	0.03	0.17	-0.05	0.06
reward lag:BPD-NON	0.00	0.03	0.00	-0.06	0.06
reward lag:HC	0.09**	0.03	3.27	0.04	0.14
RT lag:EXIT	0.01	0.01	1.26	-0.00	0.02
reward lag:EXIT	-0.00	0.01	-0.21	-0.02	0.02
RT vmax:trial	0.06**	0.02	3.19	0.02	0.10
BPD-LL:RT vmax	-0.02	0.02	-0.94	-0.05	0.02
BPD-NON:RT vmax	-0.00	0.02	-0.26	-0.04	0.03
HC:RT vmax	-0.03	0.02	-1.61	-0.06	0.01
BPD-LL:trial	-0.00	0.02	-0.14	-0.05	0.04
BPD-NON:trial	0.02	0.02	0.71	-0.03	0.06
HC:trial	-0.04	0.02	-1.61	-0.08	0.01
RT lag:trial	-0.04*	0.02	-2.07	-0.08	-0.00
RT lag:reward lag:BPD-LL	0.09***	0.03	3.53	0.04	0.14
RT lag:reward lag:BPD-NON	0.11***	0.03	3.66	0.05	0.16
RT lag:reward lag:HC	0.11***	0.03	4.29	0.06	0.16

© 2024 Tsypes A et al. *JAMA Psychiatry.*

term	coefficient	SE	t-value	95% CI low	95% CI high
**RT lag:reward lag:EXIT**	**-0.04*****	**0.01**	**-3.84**	**-0.05**	**-0.02**
BPD-LL:RT vmax:trial	-0.05*	0.03	-2.01	-0.10	-0.00
BPD-NON:RT vmax:trial	-0.01	0.03	-0.18	-0.06	0.05
HC:RT vmax:trial	-0.01	0.03	-0.36	-0.06	0.04
RT lag:BPD-LL:trial	0.03	0.03	0.96	-0.03	0.08
RT lag:BPD-NON:trial	0.04	0.03	1.23	-0.02	0.09
RT lag:HC:trial	0.01	0.03	0.29	-0.04	0.06

Note: *p<0.05; ** p<0.01; ***p<0.001. Estimates are from a multilevel linear regression model fitted to trial-level data using restricted maximum likelihood, accounting for random intercepts for each subject and run. BPD-HL (high-lethality attempters) and reward omission were the references. Dependent variable: Response time (RT) on the current trial. SE: standard error; CI: confidence interval.

RT lag: RT lagged by one trial; reward lag: Reward vs. omission feedback outcome lagged by one trial; BPD: Borderline personality disorder; BPD-LL: Low-lethality suicide attempters diagnosed with BPD (vs. high-lethality attempters diagnosed with BPD); BPD-NON: Non-attempters diagnosed with BPD (vs. high-lethality attempters diagnosed with BPD); HC: Healthy controls (vs. high-lethality attempters diagnosed with BPD); RT vmax: The most rewarded RT learned on the task to date; trial: Trial within current condition; **EXIT: Executive function**. **The bolded term represents the critical (for this sensitivity analysis) RT lag:reward lag: EXIT interaction, accounting for the effects of executive function on exploration.**

© 2024 Tsypes A et al. *JAMA Psychiatry.*

eTable 14. BPD sample sensitivity analysis: Effects of working memory

term	coefficient	SE	t-value	95% CI low	95% CI high
RT lag1	0.46***	0.01	31.41	0.43	0.49
omission lag1	-0.16***	0.02	-7.52	-0.20	-0.11
BPD-LL	0.02	0.05	0.36	-0.08	0.11
BPD-NON	-0.02	0.05	-0.30	-0.12	0.09
HC	0.00	0.05	0.10	-0.09	0.10
RT lag2	0.17***	0.02	10.84	0.14	0.20
omission lag2	-0.09***	0.02	-4.41	-0.13	-0.05
RT lag3	0.09***	0.02	5.76	0.06	0.12
omission lag3	-0.05*	0.02	-2.34	-0.09	-0.01
RT lag4	0.07***	0.02	4.48	0.04	0.10
omission lag4	0.01	0.02	0.46	-0.03	0.05
RT lag1:omission lag1	-0.43***	0.02	-22.27	-0.46	-0.39
RT lag1:BPD-LL	-0.02	0.02	-1.04	-0.06	0.02
RT lag1:BPD-NON	0.03	0.02	1.28	-0.01	0.07
RT lag1:HC	0.08***	0.02	4.31	0.04	0.12
omission lag1:BPD-LL	-0.02	0.03	-0.78	-0.07	0.03
omission lag1:BPD-NON	0.01	0.03	0.28	-0.05	0.07
omission lag1:HC	-0.09**	0.03	-3.24	-0.14	-0.03
RT lag2:omission lag2	-0.17***	0.02	-8.73	-0.21	-0.13
BPD-LL: RT lag2	-0.00	0.02	-0.08	-0.04	0.04
BPD-NON: RT lag2	0.03	0.02	1.35	-0.01	0.07
HC: RT lag2	-0.02	0.02	-0.98	-0.06	0.02
BPD-LL:omission lag2	0.07**	0.03	2.60	0.02	0.12
BPD-NON:omission lag2	0.05	0.03	1.74	-0.01	0.11
HC:omission lag2	0.04	0.03	1.36	-0.02	0.09
RT lag3:omission lag3	-0.06**	0.02	-2.91	-0.10	-0.02
BPD-LL: RT lag3	-0.05*	0.02	-2.43	-0.09	-0.01
BPD-NON: RT lag3	-0.01	0.02	-0.37	-0.05	0.04
HC:RT lag3	-0.02	0.02	-1.00	-0.06	0.02

© 2024 Tsypes A et al. *JAMA Psychiatry.*

term	coefficient	SE	t-value	95% CI low	95% CI high
BPD-LL:omission lag3	0.02	0.03	0.90	-0.03	0.08
BPD-NON:omission lag3	0.06*	0.03	2.01	0.00	0.12
HC:omission lag3	0.05	0.03	1.90	-0.00	0.10
RT lag4:omission lag4	-0.04*	0.02	-2.23	-0.08	-0.01
BPD-LL:RT lag4	0.00	0.02	0.09	-0.04	0.04
BPD-NON: RT lag4	-0.04	0.02	-1.73	-0.08	0.01
HC:RT lag4	-0.01	0.02	-0.41	-0.05	0.03
BPD-LL:omission lag4	-0.07*	0.03	-2.56	-0.12	-0.02
BPD-NON:omission lag4	-0.02	0.03	-0.63	-0.08	0.04
HC:omission lag4	0.02	0.03	0.72	-0.03	0.07
**RT lag1:omission lag1:BPD-LL**	**-0.07****	**0.03**	**-2.76**	**-0.12**	**-0.02**
**RT lag1:omission lag1:BPD-NON**	**-0.08****	**0.03**	**-2.85**	**-0.14**	**-0.03**
**RT lag1:omission lag1:HC**	**-0.10*****	**0.02**	**-4.22**	**-0.15**	**-0.06**
BPD-LL: RT lag2:omission lag2	-0.04	0.03	-1.35	-0.09	0.02
BPD-NON: RT lag2:omission lag2	-0.03	0.03	-0.97	-0.09	0.03
HC:RT lag2:omission lag2	0.01	0.03	0.45	-0.04	0.06
BPD-LL:RT lag3:omission lag3	0.03	0.03	1.26	-0.02	0.09
BPD-NON:RT lag3:omission lag3	0.01	0.03	0.38	-0.05	0.07
HC:RT lag3:omission lag3	0.01	0.03	0.22	-0.04	0.06
BPD-LL:RT lag4:omission lag4	0.01	0.03	0.33	-0.04	0.06
BPD-NON:RT lag4:omission lag4	0.05	0.03	1.58	-0.01	0.10
HC:RT lag4:omission lag4	0.01	0.03	0.41	-0.04	0.06

Note: $^{*}p<0.05$; $^{**}p<0.01$; $^{***}p<0.001$. Estimates are from a multilevel linear regression model fitted to trial-level data using restricted maximum likelihood, accounting for random intercepts for each subject and run. BPD-HL (high-lethality attempters) and reward were the references. Dependent variable: Response time (RT) on the current trial. SE: standard error; CI: confidence interval.

RT lag1: RT lagged by one trial; BPD: Borderline personality disorder; BPD-LL: Low-lethality suicide attempters diagnosed with BPD (vs. high-lethality attempters diagnosed with BPD); BPD-NON: Non-attempters diagnosed with BPD (vs. high-lethality attempters diagnosed with BPD); HC: Healthy controls (vs. high-lethality attempters diagnosed with BPD); **RT lag1-5 represents the selection buffer, and omission lag1-5 (vs. reward as reference condition), the reward buffer, and their interactions reflect assignment of rewards to choices. The RT lag2-5:Omission lag2-5:GROUP interactions test whether the buffer extends beyond one preceding trial. The bolded terms highlight that only RT lag1:omission lag1:GROUP (but not any of the RT lag2-5:Omission lag2-5:GROUP) interactions were significant, thus ruling out a working memory deficit explanation.**

© 2024 Tsypes A et al. *JAMA Psychiatry.*

© 2024 Tsypes A et al. *JAMA Psychiatry*.

eTable 15. BPD sample sensitivity analysis controlling for individual random slopes of RT lag

term	coefficient	SE	t-value	95% CI low	95% CI high
RT lag	0.31***	0.03	9.33	0.24	0.37
reward lag	0.17***	0.02	8.70	0.13	0.21
BPD-LL	0.04	0.04	0.91	-0.04	0.12
BPD-NON	0.04	0.05	0.77	-0.05	0.13
HC	0.03	0.04	0.70	-0.05	0.11
RT vmax	0.15***	0.01	12.83	0.13	0.17
trial	0.03	0.02	1.86	-0.00	0.06
RT lag:reward lag	0.44***	0.02	23.37	0.40	0.47
RT lag:BPD-LL	-0.06	0.04	-1.37	-0.14	0.03
RT lag:BPD-NON	-0.00	0.05	-0.06	-0.10	0.09
RT lag:HC	0.04	0.04	0.85	-0.05	0.12
reward lag:BPD-LL	0.00	0.03	0.19	-0.05	0.06
reward lag:BPD-NON	-0.01	0.03	-0.50	-0.07	0.04
reward lag:HC	0.08**	0.03	3.13	0.03	0.13
RT vmax:trial	0.05**	0.02	2.89	0.02	0.09
BPD-LL:RT vmax	-0.02	0.02	-1.13	-0.05	0.01
BPD-NON:RT vmax	-0.01	0.02	-0.58	-0.04	0.02
HC:RT vmax	-0.03*	0.01	-2.24	-0.06	-0.00
BPD-LL:trial	-0.00	0.02	-0.14	-0.04	0.04
BPD-NON:trial	0.01	0.02	0.26	-0.04	0.05
HC:trial	-0.05*	0.02	-2.17	-0.09	-0.00
RT lag:trial	-0.03	0.02	-1.76	-0.07	0.00
**RT lag:reward lag:BPD-LL**	**0.06***	**0.02**	**2.40**	**0.01**	**0.11**
**RT lag:reward lag:BPD-NON**	**0.08****	**0.03**	**2.76**	**0.02**	**0.13**
**RT lag:reward lag:HC**	**0.10*****	**0.02**	**4.28**	**0.06**	**0.15**
BPD-LL:RT vmax:trial	-0.03	0.02	-1.36	-0.08	0.01
BPD-NON:RT vmax:trial	0.00	0.03	0.10	-0.05	0.06
HC:RT vmax:trial	0.00	0.02	0.21	-0.04	0.05

© 2024 Tsypes A et al. *JAMA Psychiatry.*

term	coefficient	SE	t-value	95% CI low	95% CI high
RT lag:BPD-LL:trial	0.02	0.03	0.82	-0.03	0.07
RT lag:BPD-NON:trial	0.02	0.03	0.86	-0.03	0.08
RT lag:HC:trial	0.01	0.02	0.22	-0.04	0.05

Note: *p<0.05; ** p<0.01; ***p<0.001. Estimates are from a multilevel linear regression model fitted to trial-level data using restricted maximum likelihood, accounting for random intercepts for each subject and run. BPD-HL (high-lethality attempters) and reward omission were the references. Dependent variable: Response time (RT) on the current trial. SE: standard error; CI: confidence interval.

RT lag: RT lagged by one trial; reward lag: Reward vs. omission feedback outcome lagged by one trial; BPD: Borderline personality disorder; BPD-LL: Low-lethality suicide attempters diagnosed with BPD (vs. high-lethality attempters diagnosed with BPD); BPD-NON: Non-attempters diagnosed with BPD (vs. high-lethality attempters diagnosed with BPD); HC: Healthy controls (vs. high-lethality attempters diagnosed with BPD); RT vmax: The most rewarded RT learned on the task to date; trial: Trial within current condition. **The bolded terms represent critical (for this sensitivity analysis) between-group differences in exploration (RT lag:reward lag:GROUP): Group differences were not explained by individual heterogeneity of behavioral effects.**

© 2024 Tsypes A et al. *JAMA Psychiatry.*

eTable 16. BPD sample sensitivity analysis controlling for individual random slopes of RT vmax.

term	coefficient	SE	t-value	95% CI low	95% CI high
RT lag	0.28***	0.01	23.45	0.26	0.30
reward lag	0.17***	0.02	8.71	0.14	0.21
BPD-LL	0.03	0.04	0.71	-0.05	0.11
BPD-NON	0.03	0.05	0.75	-0.06	0.12
HC	0.02	0.04	0.54	-0.06	0.10
RT vmax	0.19***	0.02	9.10	0.15	0.23
trial	0.03	0.02	1.85	-0.00	0.06
RT lag:reward lag	0.44***	0.02	23.31	0.40	0.47
RT lag:BPD-LL	-0.06***	0.02	-4.13	-0.09	-0.03
RT lag:BPD-NON	-0.01	0.02	-0.76	-0.05	0.02
RT lag:HC	0.03*	0.02	2.06	0.00	0.06
reward lag:BPD-LL	0.00	0.03	0.05	-0.05	0.05
reward lag:BPD-NON	-0.02	0.03	-0.64	-0.08	0.04
reward lag:HC	0.08**	0.03	3.00	0.03	0.13
RT vmax:trial	0.06**	0.02	3.14	0.02	0.10
BPD-LL:RT vmax	-0.01	0.03	-0.54	-0.07	0.04
BPD-NON:RT vmax	-0.00	0.03	-0.10	-0.06	0.06
HC:RT vmax	-0.03	0.03	-1.28	-0.09	0.02
BPD-LL:trial	-0.00	0.02	-0.11	-0.04	0.04
BPD-NON:trial	0.01	0.02	0.41	-0.04	0.06
HC:trial	-0.05*	0.02	-2.18	-0.09	-0.00
RT lag:trial	-0.05*	0.02	-2.28	-0.09	-0.01
**RT lag:reward lag:BPD-LL**	**0.06****	**0.02**	**2.60**	**0.02**	**0.11**
**RT lag:reward lag:BPD-NON**	**0.07***	**0.03**	**2.48**	**0.01**	**0.12**
**RT lag:reward lag:HC**	**0.10*****	**0.02**	**4.27**	**0.06**	**0.15**
BPD-LL:RT vmax:trial	-0.04	0.03	-1.56	-0.09	0.01
BPD-NON:RT vmax:trial	0.00	0.03	0.10	-0.05	0.06
HC:RT vmax:trial	0.00	0.02	0.20	-0.04	0.05

© 2024 Tsypes A et al. *JAMA Psychiatry.*

term	coefficient	SE	t-value	95% CI low	95% CI high
RT lag:BPD-LL:trial	0.03	0.03	1.06	-0.02	0.08
RT lag:BPD-NON:trial	0.03	0.03	0.97	-0.03	0.08
RT lag:HC:trial	0.00	0.03	0.18	-0.04	0.05

Note: *p<0.05; ** p<0.01; ***p<0.001. Estimates are from a multilevel linear regression model fitted to trial-level data using restricted maximum likelihood, accounting for random intercepts for each subject and run. BPD-HL (high-lethality attempters) and reward omission were the references. Dependent variable: Response time (RT) on the current trial. SE: standard error; CI: confidence interval.

RT lag: RT lagged by one trial; reward lag: Reward vs. omission feedback outcome lagged by one trial; BPD: Borderline personality disorder; BPD-LL: Low-lethality suicide attempters diagnosed with BPD (vs. high-lethality attempters diagnosed with BPD); BPD-NON: Non-attempters diagnosed with BPD (vs. high-lethality attempters diagnosed with BPD); HC: Healthy controls (vs. high-lethality attempters diagnosed with BPD); RT vmax: The most rewarded RT learned on the task to date; trial: Trial within current condition. **The bolded terms represent critical (for this sensitivity analysis) between-group differences in exploration (RT lag:reward lag:GROUP): Group differences were not explained by individual heterogeneity of behavioral effects.**

© 2024 Tsypes A et al. *JAMA Psychiatry.*

eTable 17. BPD sample sensitivity analysis controlling for individual random slopes of Reward (reward lag)

term	coefficient	SE	t-value	95% CI low	95% CI high
RT lag	0.29***	0.01	24.19	0.26	0.31
reward lag	0.18***	0.04	4.46	0.10	0.26
BPD-LL	0.02	0.05	0.50	-0.07	0.12
BPD-NON	0.02	0.05	0.45	-0.08	0.13
HC	0.01	0.05	0.28	-0.08	0.11
RT vmax	0.17***	0.01	14.60	0.15	0.19
trial	0.03	0.02	1.79	-0.00	0.06
RT lag:reward lag	0.48***	0.02	25.19	0.45	0.52
RT lag:BPD-LL	-0.07***	0.02	-4.23	-0.10	-0.04
RT lag:BPD-NON	-0.02	0.02	-1.27	-0.06	0.01
RT lag:HC	0.03	0.02	1.70	-0.00	0.06
reward lag:BPD-LL	-0.00	0.05	-0.00	-0.10	0.10
reward lag:BPD-NON	-0.03	0.06	-0.51	-0.15	0.09
reward lag:HC	0.08	0.05	1.49	-0.03	0.18
RT vmax:trial	0.05*	0.02	2.49	0.01	0.09
BPD-LL:RT vmax	-0.02	0.02	-1.00	-0.05	0.01
BPD-NON:RT vmax	-0.00	0.02	-0.29	-0.04	0.03
HC:RT vmax	-0.03*	0.01	-2.04	-0.06	-0.00
BPD-LL:trial	0.00	0.02	0.15	-0.04	0.04
BPD-NON:trial	0.01	0.02	0.54	-0.03	0.06
HC:trial	-0.05*	0.02	-2.18	-0.09	-0.00
RT lag:trial	-0.04*	0.02	-2.12	-0.08	-0.00
**RT lag:reward lag:BPD-LL**	**0.06***	**0.03**	**2.22**	**0.01**	**0.11**
**RT lag:reward lag:BPD-NON**	**0.07***	**0.03**	**2.53**	**0.02**	**0.13**
**RT lag:reward lag:HC**	**0.10*****	**0.02**	**4.20**	**0.06**	**0.15**
BPD-LL:RT vmax:trial	-0.04	0.02	-1.45	-0.09	0.01
BPD-NON:RT vmax:trial	0.01	0.03	0.25	-0.05	0.06
HC:RT vmax:trial	0.01	0.02	0.28	-0.04	0.05

© 2024 Tsypes A et al. *JAMA Psychiatry.*

term	coefficient	SE	t-value	95% CI low	95% CI high
RT lag:BPD-LL:trial	0.02	0.03	0.96	-0.03	0.08
RT lag:BPD-NON:trial	0.03	0.03	0.98	-0.03	0.08
RT lag:HC:trial	0.00	0.03	0.16	-0.05	0.05

Note: *p<0.05; ** p<0.01; ***p<0.001. Estimates are from a multilevel linear regression model fitted to trial-level data using restricted maximum likelihood, accounting for random intercepts for each subject and run. BPD-HL (high-lethality attempters) and reward omission were the references. Dependent variable: Response time (RT) on the current trial. SE: standard error; CI: confidence interval.

RT lag: RT lagged by one trial; reward lag: Reward vs. omission feedback outcome lagged by one trial; BPD: Borderline personality disorder; BPD-LL: Low-lethality suicide attempters diagnosed with BPD (vs. high-lethality attempters diagnosed with BPD); BPD-NON: Non-attempters diagnosed with BPD (vs. high-lethality attempters diagnosed with BPD); HC: Healthy controls (vs. high-lethality attempters diagnosed with BPD); RT vmax: The most rewarded RT learned on the task to date; trial: Trial within current condition. **The bolded terms represent v critical (for this sensitivity analysis) between-group differences in exploration (RT lag:reward lag:GROUP): Group differences were not explained by individual heterogeneity of behavioral effects.**

© 2024 Tsypes A et al. *JAMA Psychiatry*.

eTable 18. BPD sample: model without RT vmax:Group:trial interaction as a covariate

term	coefficient	SE	t-value	95% CI low	95% CI high
RT lag	0.33***	0.01	29.39	0.31	0.35
Reward lag	0.17***	0.02	8.39	0.13	0.21
BPD-LL	0.02	0.05	0.37	-0.08	0.12
BPD-NON	0.03	0.06	0.50	-0.08	0.14
HC	0.01	0.05	0.22	-0.09	0.11
trial	0.05**	0.02	3.21	0.02	0.08
RT lag:reward lag	0.42***	0.02	22.50	0.39	0.46
RT lag:BPD-LL	-0.07***	0.01	-5.11	-0.10	-0.05
RT lag:BPD-NON	-0.01	0.02	-0.69	-0.04	0.02
RT lag:HC	0.02	0.01	1.13	-0.01	0.04
reward lag:BPD-LL	0.00	0.03	0.17	-0.05	0.06
reward lag:BPD-NON	-0.02	0.03	-0.80	-0.08	0.03
reward lag:HC	0.07**	0.03	2.71	0.02	0.12
RT lag:trial	-0.03	0.02	-1.78	-0.06	0.00
BPD-LL:trial	-0.01	0.02	-0.28	-0.05	0.03
BPD-NON:trial	0.00	0.02	0.11	-0.04	0.05
HC:trial	-0.04*	0.02	-2.09	-0.08	-0.00
**RT lag:reward lag:BPD-LL**	**0.08****	**0.03**	**3.00**	**0.03**	**0.12**
**RT lag:reward lag:BPD-NON**	**0.08****	**0.03**	**2.79**	**0.02**	**0.13**
**RT lag:reward lag:HC**	**0.11*****	**0.02**	**4.45**	**0.06**	**0.16**
RT lag:BPD-LL:trial	-0.00	0.02	-0.03	-0.04	0.04
RT lag:BPD-NON:trial	0.03	0.02	1.13	-0.02	0.08
RT lag:HC:trial	0.01	0.02	0.50	-0.03	0.05

Note: *p<0.05; ** p<0.01; ***p<0.001. Estimates are from a multilevel linear regression model fitted to trial-level data using restricted maximum likelihood, accounting for random intercepts for each subject and run. BPD-HL (high-lethality attempters) and reward omission were the references. Dependent variable: Response time (RT) on the current trial. SE: standard error; CI: confidence interval.

RT lag: RT lagged by one trial; reward lag: Reward vs. omission feedback outcome lagged by one trial; BPD: Borderline personality disorder; BPD-LL: Low-lethality suicide attempters diagnosed with BPD (vs. high-lethality attempters diagnosed with BPD); BPD-NON: Non-attempters diagnosed with BPD (vs. high-lethality attempters diagnosed with BPD); HC: Healthy controls (vs. high-lethality attempters diagnosed with BPD); trial: Trial within current condition. **The bolded terms represent**

© 2024 Tsypes A et al. *JAMA Psychiatry.*

critical (for this sensitivity analysis) between-group differences in exploration (RT lag:reward lag:GROUP) without RT vmax in the model.

© 2024 Tsypes A et al. *JAMA Psychiatry.*

eTable 19. BPD sample: Information dynamics reflective of option competition

term	coefficient	SE	t-value	95% CI low	95% CI high
BPD-LL	0.01	0.01	0.57	-0.02	0.03
BPD-NON	0.00	0.02	0.19	-0.03	0.03
HC	0.01	0.01	0.40	-0.02	0.03
trials_6-10	0.03***	0.00	8.01	0.03	0.04
trials_11-15	0.03***	0.00	6.95	0.02	0.04
trials_16-20	0.02***	0.00	5.58	0.02	0.03
trials_21-25	0.01	0.00	1.76	-0.00	0.02
trials_26-30	0.01	0.00	1.32	-0.00	0.01
trials_31-35	0.01**	0.00	2.70	0.00	0.02
trials_36-40	0.01*	0.00	2.11	0.00	0.02
**BPD-LL:trials_6-10**	**0.01***	**0.01**	**2.37**	**0.00**	**0.02**
**BPD-NON:trials_6-10**	**0.02****	**0.01**	**2.80**	**0.01**	**0.03**
**HC:trials_6-10**	**0.01***	**0.01**	**2.54**	**0.00**	**0.03**
BPD-LL:trials_11-15	0.00	0.01	0.81	-0.01	0.02
BPD-NON:trials_11-15	0.01	0.01	1.80	-0.00	0.02
HC:trials_11-15	0.01	0.01	1.61	-0.00	0.02
BPD-LL:trials_16-20	0.00	0.01	0.81	-0.01	0.02
BPD-NON:trials_16-20	0.01	0.01	1.65	-0.00	0.02
HC:trials_16-20	-0.00	0.01	-0.02	-0.01	0.01
**BPD-LL:trials_21-25**	**0.01****	**0.01**	**2.59**	**0.00**	**0.03**
**BPD-NON:trials_21-25**	**0.02*****	**0.01**	**3.33**	**0.01**	**0.03**
HC:trials_21-25	0.01	0.01	1.74	-0.00	0.02
**BPD-LL:trials_26-30**	**0.01***	**0.01**	**2.24**	**0.00**	**0.02**
BPD-NON:trials_26-30	0.01	0.01	0.84	-0.01	0.02
HC:trials_26-30	-0.00	0.01	-0.02	-0.01	0.01
BPD-LL:trials_31-35	-0.00	0.01	-0.41	-0.01	0.01
BPD-NON:trials_31-35	0.00	0.01	0.02	-0.01	0.01
HC:trials_31-35	-0.00	0.01	-0.38	-0.01	0.01
BPD-LL:trials_36-40	0.00	0.01	0.50	-0.01	0.01

© 2024 Tsypes A et al. *JAMA Psychiatry.*

term	coefficient	SE	t-value	95% CI low	95% CI high
BPD-NON:trials_36-40	-0.01	0.01	-0.92	-0.02	0.01
HC:trials_36-40	0.01	0.01	1.04	-0.01	0.02

Note: *p<0.05; ** p<0.01; ***p<0.001. Estimates are from a multilevel linear regression model fitted to trial-level data using restricted maximum likelihood, accounting for random intercepts for each subject and run. BPD-HL (high-lethality attempters) and reward omission were the references. **Dependent variable: Information dynamics (SCEPTIC model-derived entropy) on the current trial.** SE: standard error; CI: confidence interval.

BPD: Borderline personality disorder; BPD-LL: Low-lethality suicide attempters diagnosed with BPD (vs. high-lethality attempters diagnosed with BPD); BPD-NON: Non-attempters diagnosed with BPD (vs. high-lethality attempters diagnosed with BPD); HC: Healthy controls (vs. high-lethality attempters diagnosed with BPD); **trials_6-10/11-15/16-20/21-25/26-30/31-35/36-40 (vs. trials_1-5): trials 1-40 per condition binned into 5, for between-group comparisons. The bolded terms highlight that BPD-HL tended to discover fewer options than other groups, especially earlier in learning.**

© 2024 Tsypes A et al. *JAMA Psychiatry.*

eTable 20. MDD sample: Exploration and exploitation on the Clock task

term	coefficient	SE	t-value	95% CI low	95% CI high
RT lag	0.50***	0.02	23.11	0.45	0.54
reward lag	0.34***	0.04	7.51	0.25	0.42
MDD-LL	-0.11	0.08	-1.35	-0.28	0.05
MDD-SI	-0.12	0.08	-1.54	-0.28	0.03
MDD-NON	-0.14	0.08	-1.75	-0.30	0.02
HC	-0.10	0.08	-1.26	-0.25	0.06
RT vmax	0.20***	0.02	9.32	0.16	0.24
trial	0.00	0.02	0.00	-0.04	0.04
RT lag:reward lag	0.57***	0.04	14.22	0.49	0.64
RT lag:MDD-LL	-0.14***	0.03	-5.12	-0.19	-0.09
RT lag:MDD-SI	-0.06*	0.03	-2.13	-0.11	-0.00
RT lag:MDD-NON	-0.02	0.03	-0.75	-0.07	0.03
RT lag:HC	-0.06*	0.03	-2.20	-0.11	-0.01
reward lag:MDD-LL	-0.09	0.06	-1.70	-0.20	0.01
reward lag:MDD-SI	-0.16**	0.05	-3.06	-0.27	-0.06
reward lag:MDD-NON	-0.09	0.05	-1.71	-0.19	0.01
reward lag:HC	-0.15**	0.05	-3.02	-0.25	-0.05
RT vmax:trial	0.06*	0.02	2.56	0.01	0.10
MDD-LL:RT vmax	-0.00	0.03	-0.13	-0.06	0.05
MDD-SI:RT vmax	-0.00	0.03	-0.12	-0.05	0.05
MDD-NON:RT vmax	0.01	0.03	0.29	-0.04	0.06
HC:RT vmax	0.02	0.02	0.80	-0.03	0.07
MDD-LL:trial	0.00	0.03	0.18	-0.05	0.06
MDD-SI:trial	-0.01	0.03	-0.44	-0.06	0.04
MDD-NON:trial	-0.00	0.03	-0.18	-0.06	0.05
HC:trial	-0.01	0.03	-0.38	-0.06	0.04
RT lag:trial	-0.04*	0.02	-2.03	-0.08	-0.00
**RT lag:reward lag:MDD-LL**	**0.18*** **	**0.05**	**3.48**	**0.08**	**0.28**

© 2024 Tsypes A et al. *JAMA Psychiatry.*

**RT lag:reward lag:MDD-SI**	**0.08**	**0.05**	**1.68**	**-0.01**	**0.18**
**RT lag:reward lag:MDD-NON**	**0.11***	**0.05**	**2.33**	**0.02**	**0.21**
**RT lag:reward lag:HC**	**0.08**	**0.05**	**1.68**	**-0.01**	**0.17**
MDD-LL:RT vmax:trial	***-0.04***	***0.03***	***-1.49***	***-0.10***	***0.01***
MDD-SI:RT vmax:trial	***-0.04***	***0.03***	***-1.44***	***-0.10***	***0.01***
MDD-NON:RT vmax:trial	***-0.04***	***0.03***	***-1.42***	***-0.10***	***0.02***
HC:RT vmax:trial	***-0.04***	***0.03***	***-1.45***	***-0.09***	***0.01***
RT lag:MDD-LL:trial	0.04	0.03	1.60	-0.01	0.09
RT lag:MDD-SI:trial	0.04	0.03	1.68	-0.01	0.10
RT lag:MDD-NON:trial	0.03	0.03	1.09	-0.02	0.08
RT lag:HC:trial	0.00	0.02	0.08	-0.05	0.05

Note: *p<0.05; ** p<0.01; ***p<0.001. Estimates are from a multilevel linear regression model fitted to trial-level data using restricted maximum likelihood, accounting for random intercepts for each subject and run. MDD-HL (high-lethality attempters) and reward omission were the references. Dependent variable: Response time (RT) on the current trial. SE: standard error; CI: confidence interval.

RT lag: RT lagged by one trial; reward lag: Reward vs. omission feedback outcome lagged by one trial; MDD: Major depressive disorder; MDD-LL: Low-lethality suicide attempters diagnosed with MDD(vs. high-lethality attempters diagnosed with MDD); MDD-SI: Non-attempters with suicidal ideation diagnosed with MDD; MDD-NON: Non-attempters diagnosed with MDD (vs. high-lethality attempters diagnosed with MDD); HC: Healthy controls (vs. high-lethality attempters diagnosed with MDD); RT vmax: The most rewarded RT learned on the task to date; trial: Trial within current condition. **The bolded terms represent between-group differences in exploration (RT lag:reward lag:GROUP) and exploitation** (***GROUP:RT vmax:trial***).

© 2024 Tsypes A et al. *JAMA Psychiatry.*

eTable 21. BPD sample: model without Reward lag:RT lag interaction as a covariate

term	coefficient	SE	t-value	95% CI low	95% CI high
RT lag	0.27***	0.01	23.69	0.25	0.30
BPD-LL	0.01	0.05	0.21	-0.08	0.10
BPD-NON	0.01	0.05	0.29	-0.08	0.11
HC	-0.00	0.04	-0.02	-0.09	0.09
RT vmax	0.17***	0.01	13.42	0.14	0.19
RT lag:BPD-LL	-0.07***	0.02	-4.38	-0.10	-0.04
RT lag:BPD-NON	0.01	0.02	0.57	-0.02	0.04
RT lag:HC	0.02	0.01	1.51	-0.01	0.05
BPD-LL:RT vmax	***-0.02***	***0.02***	***-1.03***	***-0.05***	***0.02***
BPD-NON:RT vmax	***-0.01***	***0.02***	***-0.33***	***-0.04***	***0.03***
HC:RT vmax	***-0.04*****	***0.02***	***-2.69***	***-0.07***	***-0.01***

Note: *p<0.05; ** p<0.01; ***p<0.001. Estimates are from a multilevel linear regression model fitted to trial-level data using restricted maximum likelihood, accounting for random intercepts for each subject and run. BPD-HL (high-lethality attempters) and reward omission were the references. Dependent variable: Response time (RT) on the current trial. SE: standard error; CI: confidence interval.

RT lag: RT lagged by one trial; reward lag: Reward vs. omission feedback outcome lagged by one trial; BPD: Borderline personality disorder; BPD-LL: Low-lethality suicide attempters diagnosed with BPD (vs. high-lethality attempters diagnosed with BPD); BPD-NON: Non-attempters diagnosed with BPD (vs. high-lethality attempters diagnosed with BPD); HC: Healthy controls (vs. high-lethality attempters diagnosed with BPD); RT vmax: The most rewarded RT learned on the task to date. **The bolded terms represent between-group differences in overall exploitation (GROUP:RT vmax) without over-controlling by partialing out the effects of Reward lag:RT lag interaction.**

© 2024 Tsypes A et al. *JAMA Psychiatry.*

eTable 22. BPD EMA subsample: Exploration and exploitation on the Clock task predicting the frequency of prospective daily suicidal ideation.

term	coefficient	SE	t-value	95% CI low	95% CI high
RT lag	0.25***	0.01	32.79	0.24	0.26
reward lag	0.17***	0.01	13.56	0.15	0.20
SI	-0.01	0.02	-0.50	-0.04	0.03
RT vmax	0.17***	0.01	22.86	0.16	0.19
trial	0.03*	0.01	2.51	0.01	0.05
RT lag:reward lag	0.50***	0.01	40.02	0.48	0.53
RT lag:SI	0.04***	0.01	5.49	0.03	0.06
reward lag:SI	-0.02	0.01	-1.78	-0.05	0.00
RT vmax:trial	0.04**	0.01	3.11	0.01	0.06
SI:RT vmax	-0.02	0.01	-1.91	-0.03	0.00
SI:trial	0.01	0.01	0.98	-0.01	0.03
RT lag:trial	-0.02	0.01	-1.51	-0.04	0.01
**RT lag:reward lag:SI**	**-0.04*** **	**0.01**	**-3.36**	**-0.07**	**-0.02**
SI:RT vmax:trial	***0.01***	***0.01***	***0.97***	***-0.01***	***0.04***
RT lag:SI:trial	-0.01	0.01	-0.60	-0.03	0.02

Note: *p<0.05; ** p<0.01; ***p<0.001. Estimates are from a multilevel linear regression model fitted to trial-level data using restricted maximum likelihood, accounting for random intercepts for each subject and run. Reward omission was the reference. Dependent variable: Response time (RT) on the current trial. SE: standard error; CI: confidence interval.

RT lag: RT lagged by one trial; reward lag: Reward vs. omission feedback outcome lagged by one trial; SI: suicidal ideation; RT vmax: The most rewarded RT learned on the task to date; trial: Trial within current condition. **The bolded terms represent differences in exploration (RT lag:reward lag:SI) and exploitation (*SI:RT vmax:trial*) based on the frequency of prospective suicidal ideation.**

eTable 23. BPD EMA subsample sensitivity analysis: Excluding 2 relatively extreme values on daily suicidal ideation measure

term	coefficient	SE	t-value	95% CI low	95% CI high
RT lag	0.25***	0.01	32.23	0.23	0.26
reward lag	0.18***	0.01	13.50	0.15	0.20
SI	0.01	0.02	0.48	-0.03	0.04
RT vmax	0.18***	0.01	22.68	0.16	0.19
trial	0.02*	0.01	2.26	0.00	0.04
RT lag:reward lag	0.51***	0.01	39.94	0.48	0.53
**RT lag:SI**	**0.05*** **	**0.01**	**6.29**	**0.03**	**0.07**
reward lag:SI	-0.02	0.01	-1.53	-0.05	0.01
RT vmax:trial	0.04**	0.01	3.26	0.02	0.06
SI:RT vmax	-0.01	0.01	-1.56	-0.03	0.00
SI:trial	0.00	0.01	0.16	-0.02	0.02
RT lag:trial	-0.02	0.01	-1.61	-0.05	0.00
**RT lag:reward lag:SI**	**-0.04** **	**0.01**	**-3.00**	**-0.06**	**-0.01**
SI:RT vmax:trial	***0.03* ***	***0.01***	***2.07***	***0.00***	***0.05***
RT lag:SI:trial	-0.01	0.01	-0.97	-0.04	0.01

Note: *p<0.05; ** p<0.01; ***p<0.001. Estimates are from a multilevel linear regression model fitted to trial-level data using restricted maximum likelihood, accounting for random intercepts for each subject and run. Reward omission was the reference. Dependent variable: Response time (RT) on the current trial. SE: standard error; CI: confidence interval.

RT lag: RT lagged by one trial; reward lag: Reward vs. omission feedback outcome lagged by one trial; SI: suicidal ideation; RT vmax: The most rewarded RT learned on the task to date; trial: Trial within current condition. **The bolded terms represent differences in exploration (RT lag:reward lag:SI) and exploitation (*SI:RT vmax:trial*) based on the frequency of prospective suicidal ideation.**

© 2024 Tsypes A et al. *JAMA Psychiatry.*

eTable 24. BPD EMA subsample sensitivity analysis: SA recency

term	coefficient	SE	t-value	95% CI low	95% CI high
RT lag	0.25***	0.01	27.10	0.23	0.27
reward lag	0.17***	0.02	10.71	0.14	0.20
SI	-0.02	0.02	-0.96	-0.06	0.02
SA recency	-0.03	0.02	-1.26	-0.08	0.02
RT vmax	0.17***	0.01	17.96	0.15	0.19
trial	0.02*	0.01	1.96	0.00	0.05
RT lag:reward lag	0.50***	0.02	32.37	0.47	0.53
RT lag:SI	0.04***	0.01	4.99	0.02	0.06
reward lag:SI	-0.02	0.01	-1.13	-0.04	0.01
RT lag:SA recency	-0.01	0.01	-1.48	-0.03	0.00
reward lag:SA recency	0.03	0.02	1.66	-0.00	0.06
RT vmax:trial	0.03	0.02	1.68	-0.00	0.05
SI:RT vmax	-0.02	0.01	-1.80	-0.03	0.00
SI:trial	0.01	0.01	0.86	-0.01	0.03
RT lag:trial	-0.02	0.02	-1.04	-0.05	0.01
RT lag:reward lag:SI	-0.03*	0.01	-2.39	-0.06	-0.01
**RT lag:reward lag:SA recency**	**0.04***	**0.02**	**2.47**	**0.01**	**0.07**
SI:RT vmax:trial	0.01	0.01	0.99	-0.01	0.04
RT lag:SI:trial	-0.01	0.01	-0.62	-0.03	0.02

Note: *p<0.05; ** p<0.01; ***p<0.001. Estimates are from a multilevel linear regression model fitted to trial-level data using restricted maximum likelihood, accounting for random intercepts for each subject and run. Reward omission was the reference. Dependent variable: Response time (RT) on the current trial. SE: standard error; CI: confidence interval.

RT lag: RT lagged by one trial; reward lag: Reward vs. omission feedback outcome lagged by one trial; SI: suicidal ideation; RT vmax: The most rewarded RT learned on the task to date; trial: Trial within current condition; **SA recency: Time between the most recent suicide attempt and Clock task completion. The bolded term represents the critical (for this sensitivity analysis) RT lag:reward lag:SA recency interaction, accounting for the effects of suicide attempt recency on exploration.**

© 2024 Tsypes A et al. *JAMA Psychiatry.*

eTable 25. BPD EMA subsample sensitivity analysis: Controlling for average levels of negative internalizing affect in daily life

term	coefficient	SE	t-value	95% CI low	95% CI high
RT lag	0.25***	0.01	32.84	0.24	0.27
reward lag	0.18***	0.01	13.67	0.15	0.20
SI	-0.01	0.02	-0.78	-0.05	0.02
int	0.03	0.02	1.67	-0.01	0.06
RT vmax	0.17***	0.01	22.83	0.16	0.19
trial	0.03*	0.01	2.52	0.01	0.05
RT lag:reward lag	0.51***	0.01	40.01	0.48	0.53
RT lag:SI	0.04***	0.01	5.41	0.03	0.06
reward lag:SI	-0.02	0.01	-1.48	-0.05	0.01
RT lag:int	-0.00	0.01	-0.56	-0.02	0.01
reward lag:int	-0.02	0.01	-1.41	-0.04	0.01
RT vmax:trial	0.04**	0.01	3.08	0.01	0.06
SI:RT vmax	-0.02	0.01	-1.91	-0.03	0.00
SI:trial	0.01	0.01	1.00	-0.01	0.03
RT lag:trial	-0.02	0.01	-1.51	-0.04	0.01
RT lag:reward lag:SI	-0.04**	0.01	-2.87	-0.07	-0.01
**RT lag:reward lag:int**	**-0.03***	**0.01**	**-2.16**	**-0.05**	**-0.00**
SI:RT vmax:trial	0.01	0.01	0.89	-0.01	0.03
RT lag:SI:trial	-0.01	0.01	-0.62	-0.03	0.02

Note: *p<0.05; ** p<0.01; ***p<0.001. Estimates are from a multilevel linear regression model fitted to trial-level data using restricted maximum likelihood, accounting for random intercepts for each subject and run. Reward omission was the reference. Dependent variable: Response time (RT) on the current trial. SE: standard error; CI: confidence interval.

RT lag: RT lagged by one trial; reward lag: Reward vs. omission feedback outcome lagged by one trial; SI: suicidal ideation; RT vmax: The most rewarded RT learned on the task to date; trial: Trial within current condition; **int: mean levels of internalizing affect over the duration of the EMA protocol (see supplemental methods: Daily assessments of affect for more details). The bolded term represents the critical (for this sensitivity analysis) RT lag:reward lag:int interaction, accounting for the effects of daily internalizing affect on exploration.**

© 2024 Tsypes A et al. *JAMA Psychiatry.*

eTable 26. BPD EMA subsample sensitivity analysis: Controlling for average levels of externalizing affect in daily life

term	coefficient	SE	t-value	95% CI low	95% CI high
RT lag	0.25***	0.01	32.78	0.24	0.26
reward lag	0.17***	0.01	13.53	0.15	0.20
SI	-0.01	0.02	-0.46	-0.04	0.03
ext	0.01	0.02	0.81	-0.02	0.05
RT vmax	0.17***	0.01	22.77	0.16	0.19
trial	0.03*	0.01	2.55	0.01	0.05
RT lag:reward lag	0.51***	0.01	40.09	0.48	0.53
RT lag:SI	0.04***	0.01	5.63	0.03	0.06
reward lag:SI	-0.02	0.01	-1.81	-0.05	0.00
RT lag:ext	-0.03***	0.01	-4.46	-0.04	-0.02
reward lag:ext	-0.00	0.01	-0.21	-0.03	0.02
RT vmax:trial	0.04**	0.01	3.15	0.01	0.06
SI:RT vmax	-0.02	0.01	-1.90	-0.03	0.00
SI:trial	0.01	0.01	1.04	-0.01	0.03
RT lag:trial	-0.02	0.01	-1.53	-0.04	0.01
RT lag:reward lag:SI	-0.05***	0.01	-3.51	-0.07	-0.02
**RT lag:reward lag:ext**	**0.03***	**0.01**	**2.32**	**0.00**	**0.05**
SI:RT vmax:trial	0.01	0.01	0.98	-0.01	0.04
RT lag:SI:trial	-0.01	0.01	-0.53	-0.03	0.02

Note: *p<0.05; ** p<0.01; ***p<0.001. Estimates are from a multilevel linear regression model fitted to trial-level data using restricted maximum likelihood, accounting for random intercepts for each subject and run. Reward omission was the reference. Dependent variable: Response time (RT) on the current trial. SE: standard error; CI: confidence interval.

RT lag: RT lagged by one trial; reward lag: Reward vs. omission feedback outcome lagged by one trial; SI: suicidal ideation; RT vmax: The most rewarded RT learned on the task to date; trial: Trial within current condition; **ext: mean levels of externalizing affect over the duration of the EMA protocol (see supplemental methods: Daily assessments of affect for more details). The bolded term represents the critical (for this sensitivity analysis) RT lag:reward lag:ext interaction, accounting for the effects of daily externalizing affect on exploration.**

© 2024 Tsypes A et al. *JAMA Psychiatry.*

eTable 27. BPD EMA subsample sensitivity analysis: Controlling for average levels of impulsive affect in daily life

term	coefficient	SE	t-value	95% CI low	95% CI high
RT lag	0.25***	0.01	32.92	0.24	0.27
reward lag	0.18***	0.01	13.62	0.15	0.20
SI	-0.01	0.02	-0.75	-0.05	0.02
imp	0.03	0.02	1.43	-0.01	0.06
RT vmax	0.17***	0.01	22.84	0.16	0.19
trial	0.03*	0.01	2.57	0.01	0.05
RT lag:reward lag	0.51***	0.01	40.16	0.48	0.53
RT lag:SI	0.05***	0.01	6.14	0.03	0.06
reward lag:SI	-0.02	0.01	-1.50	-0.05	0.01
RT lag:imp	-0.03***	0.01	-4.02	-0.04	-0.01
reward lag:imp	-0.02	0.01	-1.25	-0.04	0.01
RT vmax:trial	0.04**	0.01	3.14	0.01	0.06
SI:RT vmax	-0.02	0.01	-1.88	-0.03	0.00
SI:trial	0.01	0.01	1.06	-0.01	0.03
RT lag:trial	-0.02	0.01	-1.53	-0.04	0.01
RT lag:reward lag:SI	-0.04***	0.01	-3.30	-0.07	-0.02
**RT lag:reward lag:imp**	**-0.00**	**0.01**	**-0.34**	**-0.03**	**0.02**
SI:RT vmax:trial	0.01	0.01	0.93	-0.01	0.03
RT lag:SI:trial	-0.01	0.01	-0.55	-0.03	0.02

Note: *p<0.05; ** p<0.01; ***p<0.001. Estimates are from a multilevel linear regression model fitted to trial-level data using restricted maximum likelihood, accounting for random intercepts for each subject and run. Reward omission was the reference. Dependent variable: Response time (RT) on the current trial. SE: standard error; CI: confidence interval.

RT lag: RT lagged by one trial; reward lag: Reward vs. omission feedback outcome lagged by one trial; SI: suicidal ideation; RT vmax: The most rewarded RT learned on the task to date; trial: Trial within current condition; **imp: mean levels of impulsive affect over the duration of the EMA protocol (see supplemental methods: Daily assessments of affect for more details). The bolded term represents the critical (for this sensitivity analysis) RT lag:reward lag:imp interaction, accounting for the effects of daily impulsive affect on exploration.**

© 2024 Tsypes A et al. *JAMA Psychiatry.*

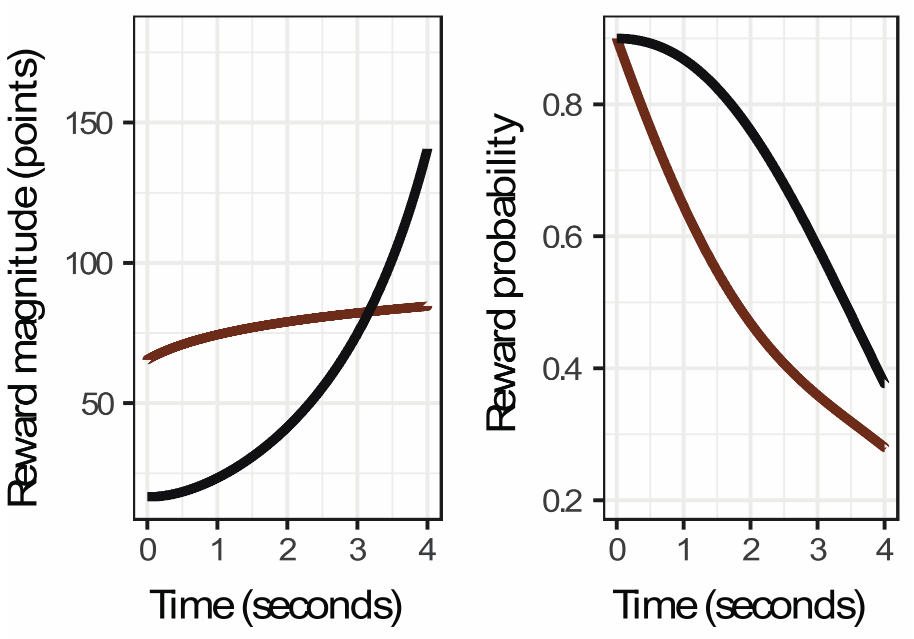

eFigure 1. Reward magnitude and probability across time-varying contingencies
Reward magnitude and probability varied independently across two time-varying contingencies: Increasing expected value, IEV, in black color, and decreasing expected value, DEV, in brown color.

© 2024 Tsypes A et al. *JAMA Psychiatry.*

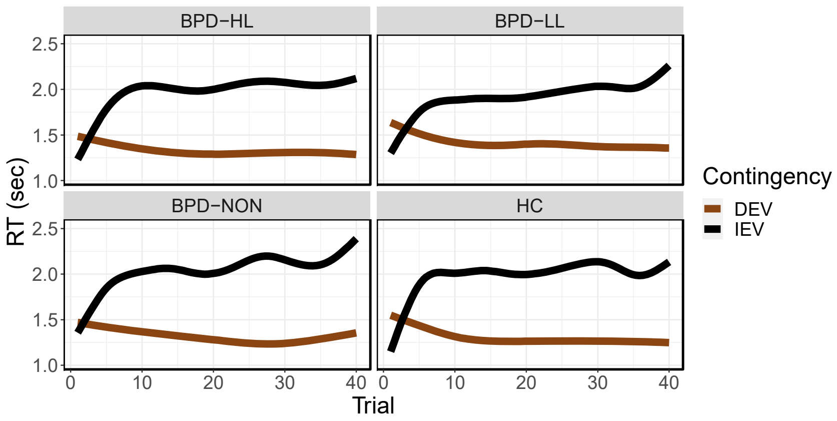

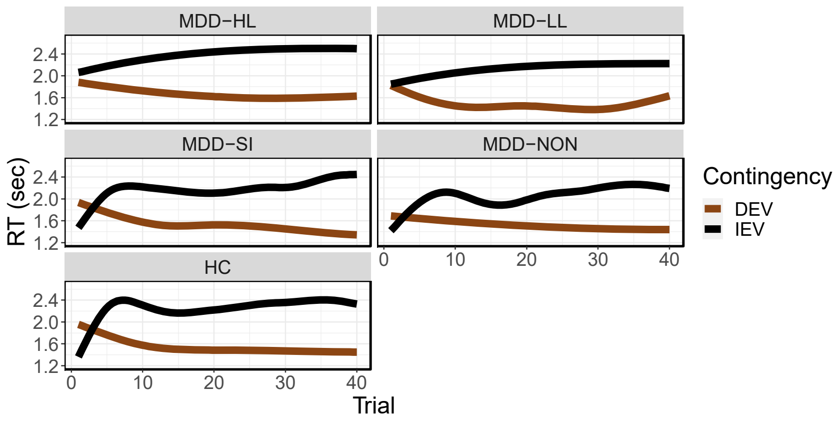

eFigure 2. Behavioral manipulation checks by group (generalized additive model)
Evolution of participants' response times (RT) by contingency in the borderline personality disorder (BPD, left) and major depressive disorder (MDD, right) samples. Increasing expected value: IEV, black color, and decreasing expected value: DEV, brown color. The shaded gray area around the lines represents a 95% confidence interval. Plotted data are smoothed using a Generalized Additive Model (GAM) in *ggplot2* R package. In plots on the right, the smoothing employed natural splines from the *splines* R package, with a basis of 5 knots. The difference between DEV and IEV at trial 1 is due to the alternation of IEV and DEV conditions, which change every 40 trials of the task. HL: high-lethality; LL: low-lethality; NON-non-attempters; HC: healthy controls; SI: ideators.

© 2024 Tsypes A et al. *JAMA Psychiatry.*

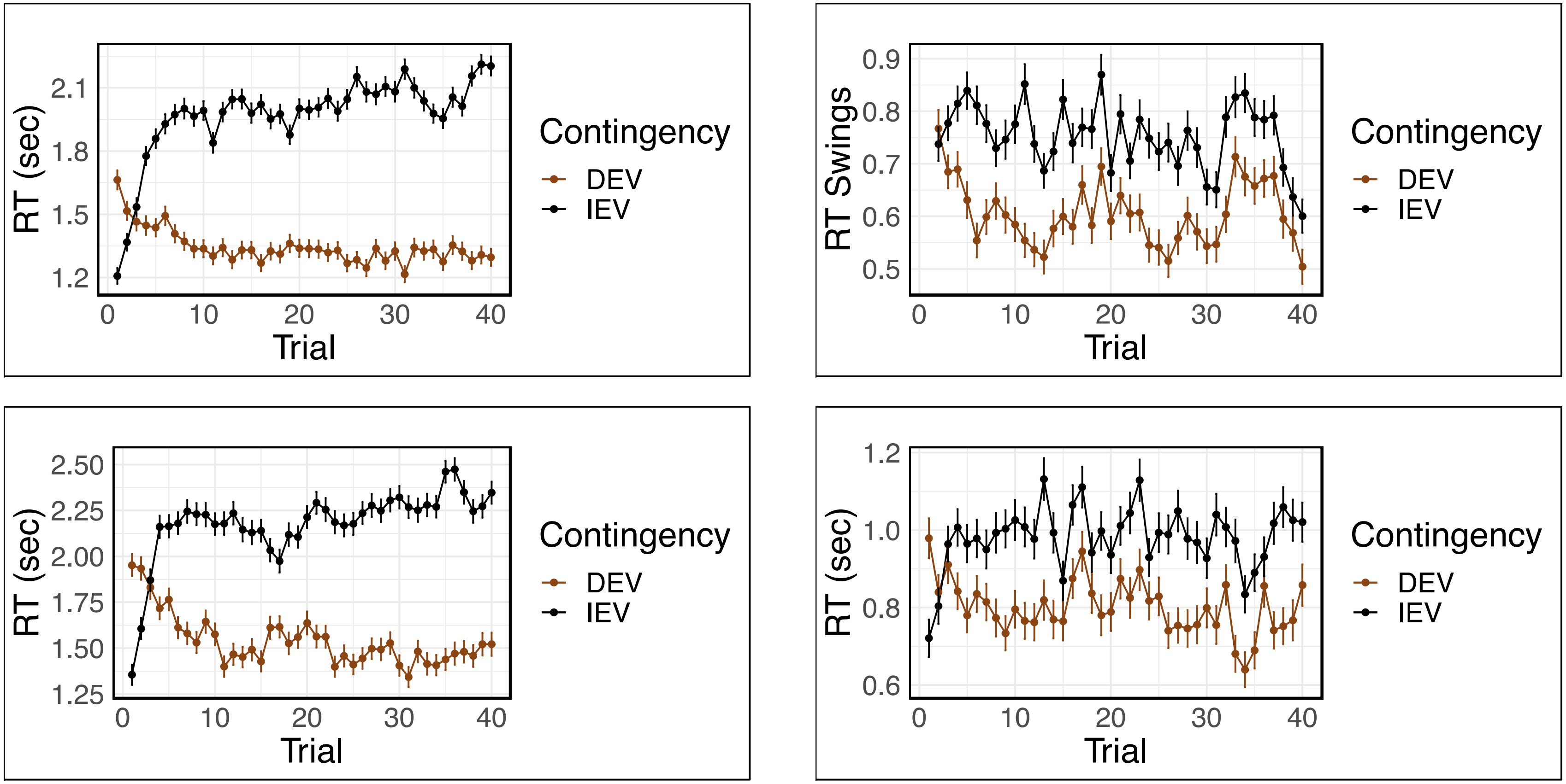

eFigure 3. Behavioral manipulation checks (raw data, trial averages across groups)
Evolution of participants' response times (RT, left) and RT swings (right) by contingency in the BPD (top panel) and MDD (bottom panel) samples. Increasing expected value: IEV, black color, and decreasing expected value: DEV, brown color. The difference between DEV and IEV at trial 1 is due to the alternation of IEV and DEV conditions, which change every 40 trials of the task.

© 2024 Tsypes A et al. *JAMA Psychiatry.*

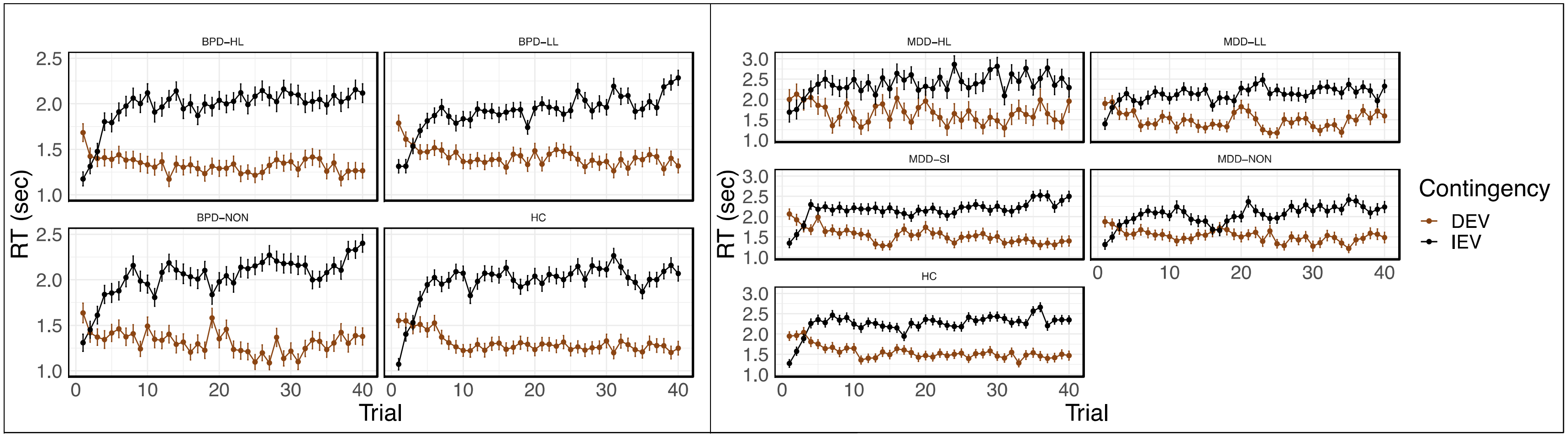

eFigure 4. Behavioral manipulation checks (raw data, trial averages by group)
Evolution of participants' response times (RT) by contingency in the BPD (left panel) and MDD (right panel) samples. Increasing expected value: IEV, black color, and decreasing expected value: DEV, brown color. The difference between DEV and IEV at trial 1 is due to the alternation of IEV and DEV conditions, which change every 40 trials of the task. HL: high-lethality; LL: low-lethality; NON-non-attempters; HC: healthy controls; SI: ideators.

© 2024 Tsypes A et al. *JAMA Psychiatry.*

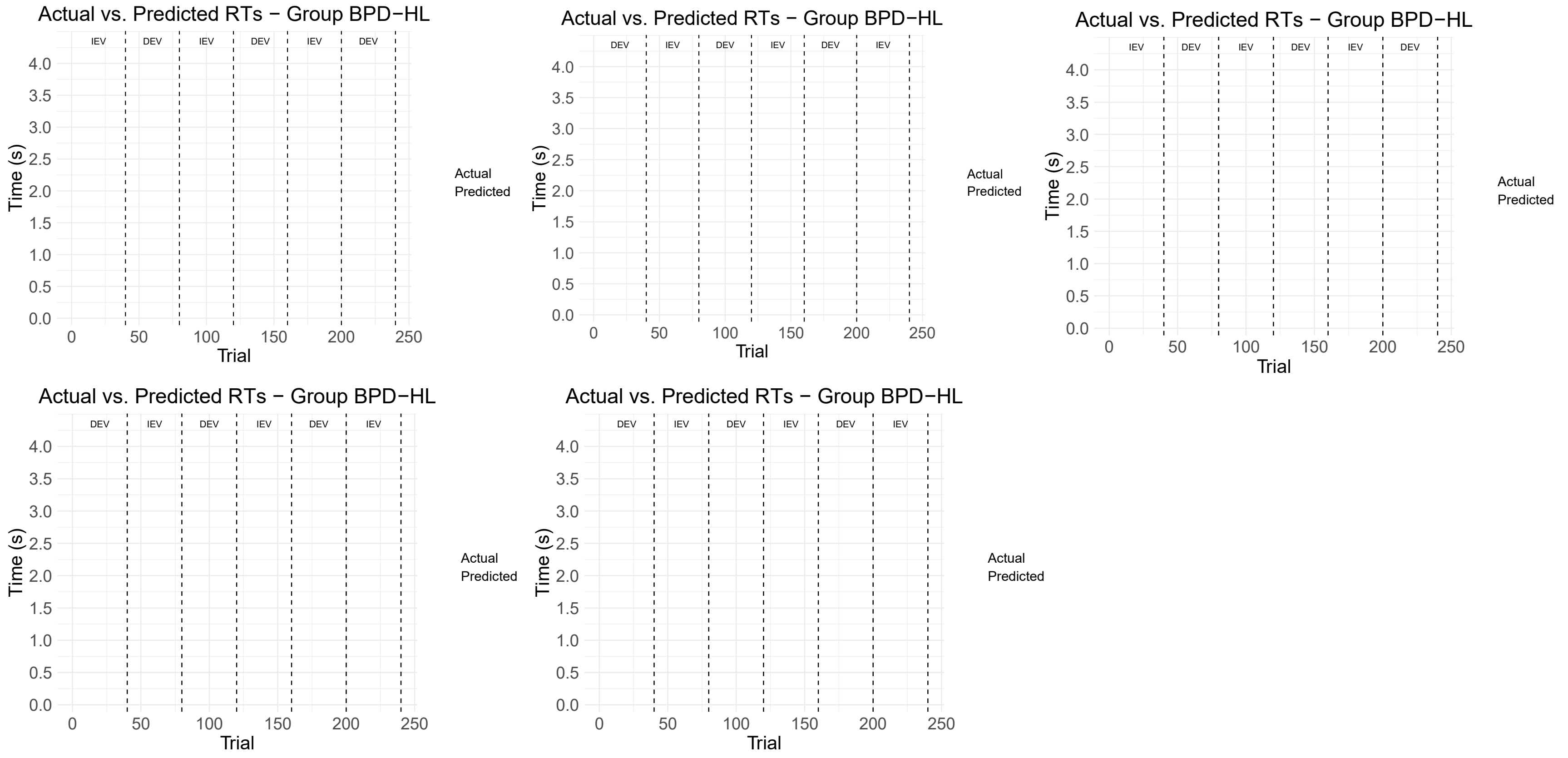

eFigure 5a. Posterior Predictive Checks: Borderline personality disorder diagnosis, high-lethality attempters (BPD-HL)
Trial-by-trial fits of the information-compressing RL SCEPTIC model variant in five randomly selected BPD-HL participants at minimally informative prior values of the parameters. Participants' actual response times (RTs) are depicted in green, whereas their model-predicted most probable RTs are depicted in orange. The response variable is distributed multinomially across 40 bins. To simplify visualization, only the location of model-predicted RT_{Vmax} (exploitative responses) is depicted. Thus, these plots do not reflect SCEPTIC predictions concerning exploration (RT swings). Please see examples of full posterior predictive checks in[7]. At the prior parameter values, individual fits are qualitatively the same, indicating that fits are not achieved by overfitting.

© 2024 Tsypes A et al. *JAMA Psychiatry.*

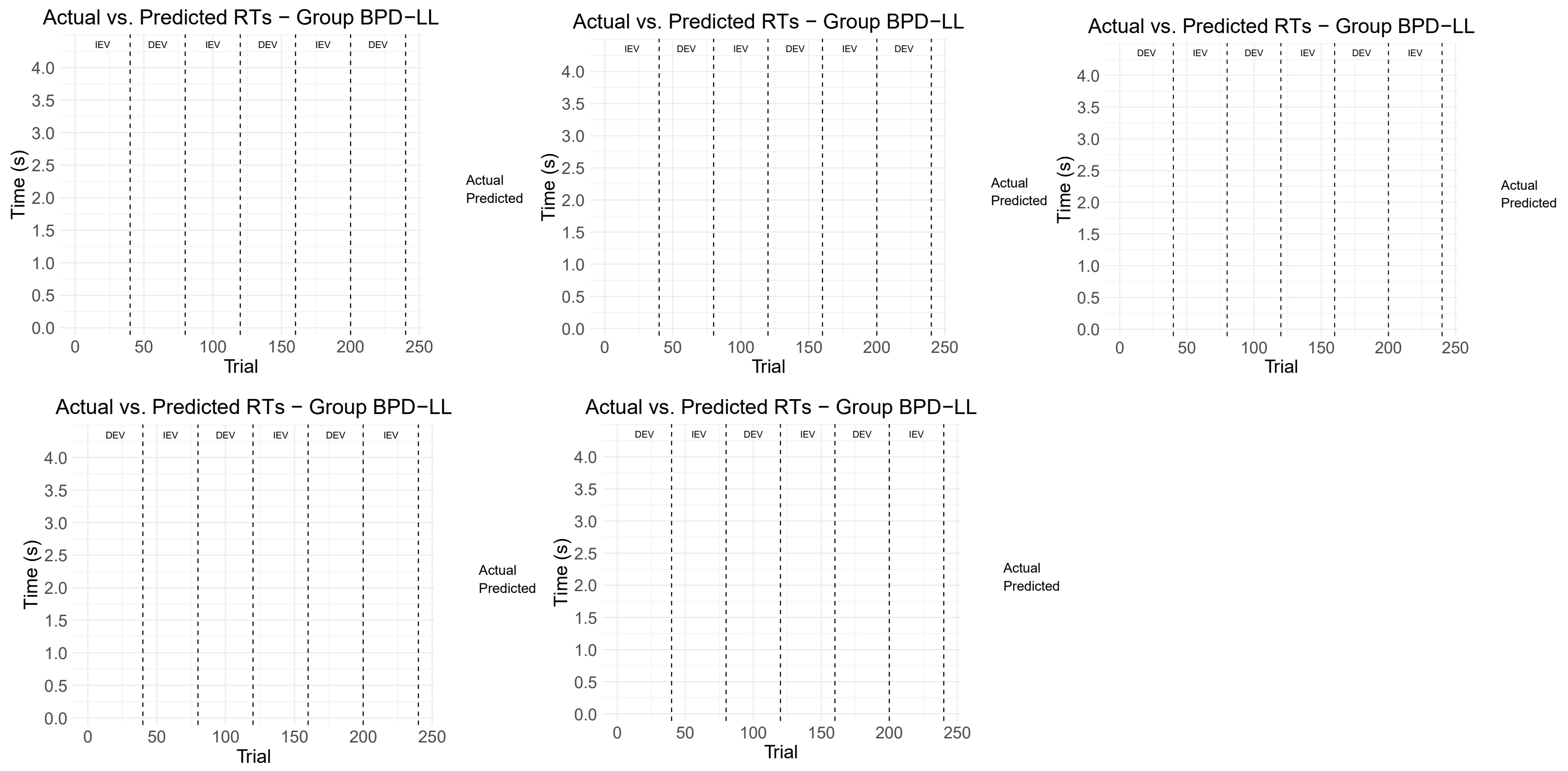

eFigure 5b. Posterior Predictive Checks: Borderline personality disorder diagnosis, low-lethality attempters (BPD-LL)
Trial-by-trial fits of the information-compressing RL SCEPTIC model variant in five randomly selected BPD-LL participants at minimally informative prior values of the parameters. Participants' actual response times (RTs) are depicted in green, whereas their model-predicted most probable RTs are depicted in orange. The response variable is distributed multinomially across 40 bins. To simplify visualization, only the location of model-predicted RT_{Vmax} (exploitative responses) is depicted. Thus, these plots do not reflect SCEPTIC predictions concerning exploration (RT swings). Please see examples of full posterior predictive checks in[7]. At the prior parameter values, individual fits are qualitatively the same, indicating that fits are not achieved by overfitting.

© 2024 Tsypes A et al. *JAMA Psychiatry.*

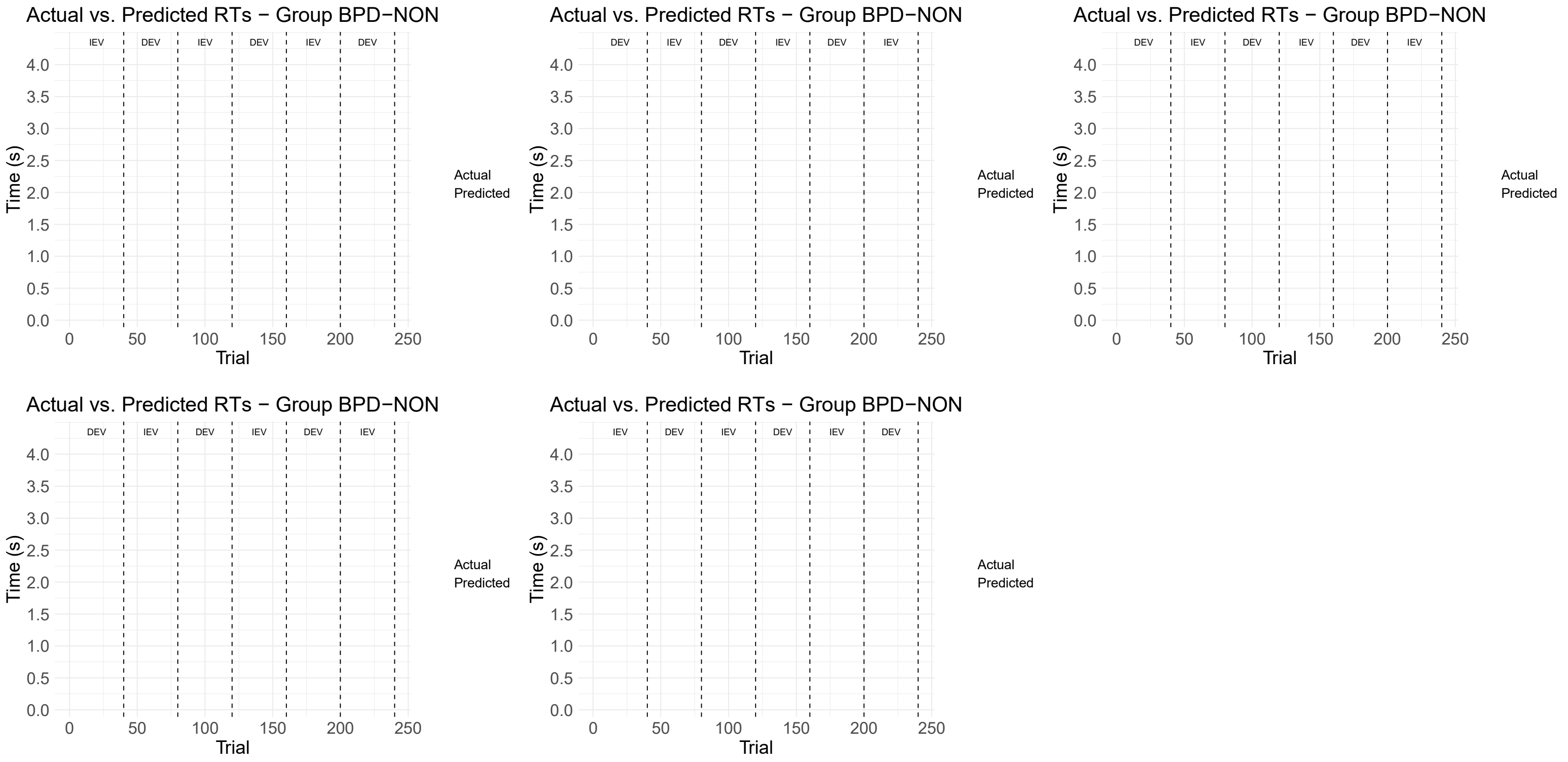

eFigure 5c. Posterior Predictive Checks: Borderline personality disorder diagnosis, non-attempters (BPD-NON)
Trial-by-trial fits of the information-compressing RL SCEPTIC model variant in five randomly selected BPD-NON participants at minimally informative prior values of the parameters. Participants' actual response times (RTs) are depicted in green, whereas their model-predicted most probable RTs are depicted in orange. The response variable is distributed multinomially across 40 bins. To simplify visualization, only the location of model-predicted RT_{Vmax} (exploitative responses) is depicted. Thus, these plots do not reflect SCEPTIC predictions concerning exploration (RT swings). Please see examples of full posterior predictive checks in[7]. At the prior parameter values, individual fits are qualitatively the same, indicating that fits are not achieved by overfitting.

© 2024 Tsypes A et al. *JAMA Psychiatry.*

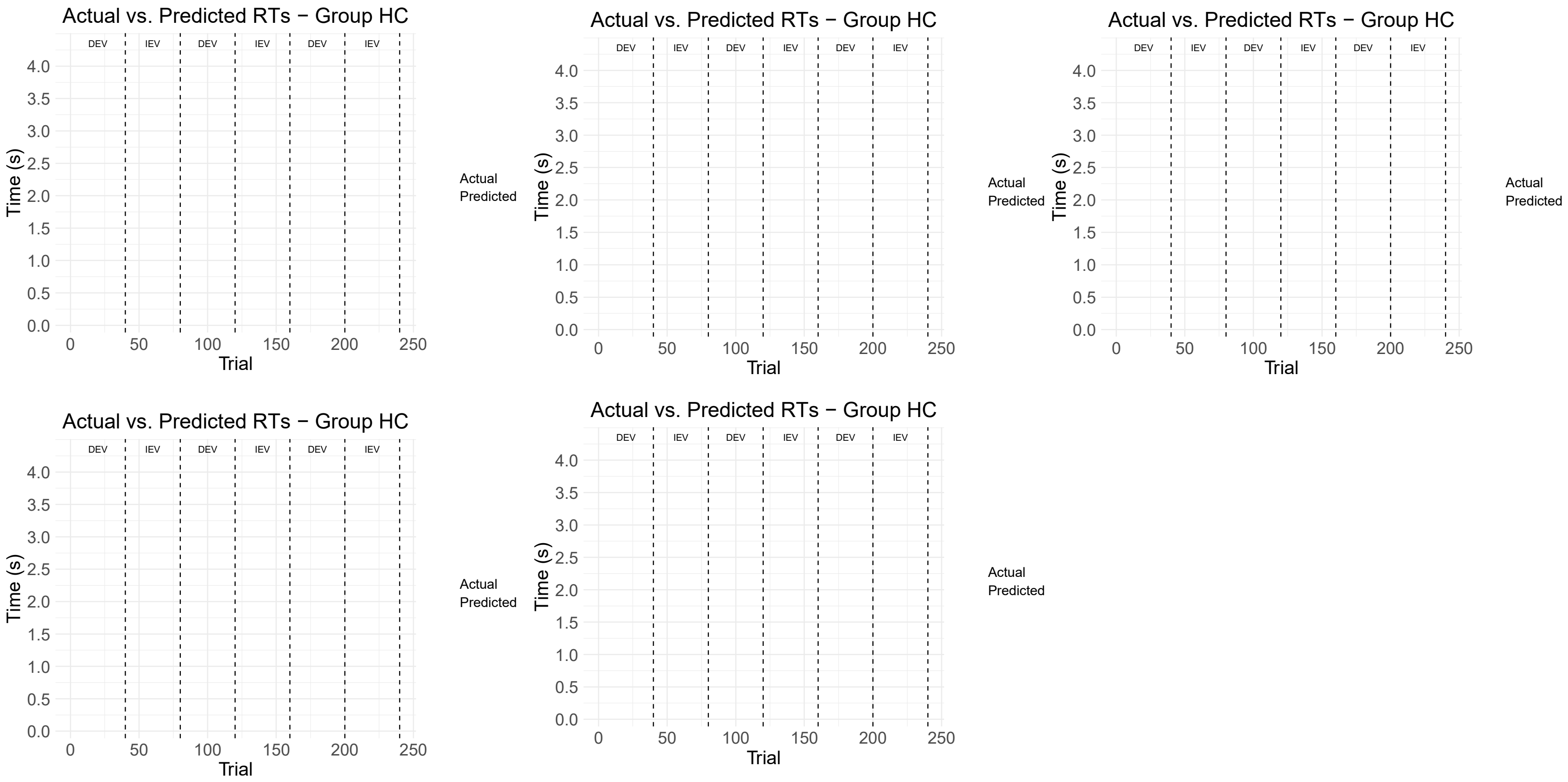

eFigure 5d. Posterior Predictive Checks: Healthy controls (HC)
Trial-by-trial fits of the information-compressing RL SCEPTIC model variant in five randomly selected HC participants at minimally informative prior values of the parameters. Participants' actual response times (RTs) are depicted in green, whereas their model-predicted most probable RTs are depicted in orange. The response variable is distributed multinomially across 40 bins. To simplify visualization, only the location of model-predicted RT_{Vmax} (exploitative responses) is depicted. Thus, these plots do not reflect SCEPTIC predictions concerning exploration (RT swings). Please see examples of full posterior predictive checks in[7]. At the prior parameter values, individual fits are qualitatively the same, indicating that fits are not achieved by overfitting.

© 2024 Tsypes A et al. *JAMA Psychiatry.*
