# Supplementary material for: Exploration-Exploitation and Suicidal Behavior in Borderline Personality Disorder and Depression
Source: JAMA Psychiatry. 2024 Jul 10;81(10):1010–9. doi: 10.1001/jamapsychiatry.2024.1796 (PMC11238070; doi:10.1001/jamapsychiatry.2024.1796)
Supplement: Supplement 2. — Data sharing statement [file jamapsychiatry-e241796-s002.pdf]

Data Sharing Statement

Tsypes. Exploration-Exploitation and Suicidal Behavior in Borderline Personality Disorder and Depression. *JAMA Psychiatry*. Published July 10, 2024. doi:10.1001/jamapsychiatry.2024.1796

Data
Data available: Yes
Data types: Deidentified participant data, Data dictionary
How to access data: https://github.com/tsypesa/Explore_Exploit_SB
When available: With publication

Supporting Documents
Document types: Statistical/analytic code
How to access documents: https://github.com/tsypesa/Explore_Exploit_SB
When available: With publication

Additional Information
Who can access the data: Anyone requesting the data.
Types of analyses: To evaluate key analyses in the paper.
Mechanisms of data availability: With investigator support.
